# One A_3_B Porphyrin Structure—Three Successful Applications

**DOI:** 10.3390/nano12111930

**Published:** 2022-06-05

**Authors:** Ion Fratilescu, Anca Lascu, Bogdan Ovidiu Taranu, Camelia Epuran, Mihaela Birdeanu, Ana-Maria Macsim, Eugenia Tanasa, Eugeniu Vasile, Eugenia Fagadar-Cosma

**Affiliations:** 1 Institute of Chemistry “Coriolan Dragulescu”, Mihai Viteazu Ave. 24, 300223 Timisoara, Romania; ionfratilescu@acad-icht.tm.edu.ro (I.F.); alascu@acad-icht.tm.edu.ro (A.L.); ecamelia@acad-icht.tm.edu.ro (C.E.); 2 National Institute for Research and Development in Electrochemistry and Condensed Matter, Plautius Andronescu Street 1, 300224 Timisoara, Romania; mihaelabirdeanu@gmail.com; 3 Institute of Macromolecular Chemistry “Petru Poni”, Grigore Ghica Vodă Alley, No. 41A, 700487 Iasi, Romania; macsim.ana@icmpp.ro; 4 Faculty of Applied Chemistry and Materials Science, University Politehnica of Bucharest, Splaiul Independentei 313, Sector 6, 060042 Bucharest, Romania; eugenia.vasile27@gmail.com (E.T.); eugeniu.vasile@upb.ro (E.V.)

**Keywords:** A_3_B porphyrin, Pt-metalloporphyrin, NMR, UV-Vis, fluorescence, AFM, TEM, SEM microscopy, hydroquinone detection, steel corrosion inhibition, HER and OER electrocatalytic activity

## Abstract

Porphyrins are versatile structures capable of acting in multiple ways. A mixed substituted A_3_B porphyrin, 5-(3-hydroxy-phenyl)-10,15,20-tris-(3-methoxy-phenyl)-porphyrin and its Pt(II) complex, were synthesised and fully characterised by ^1^H- and ^13^C-NMR, TLC, UV-Vis, FT-IR, fluorescence, AFM, TEM and SEM with EDX microscopy, both in organic solvents and in acidic mediums. The pure compounds were used, firstly, as sensitive materials for sensitive and selective optical and fluorescence detection of hydroquinone with the best results in the range 0.039–6.71 µM and a detection limit of 0.013 µM and, secondly, as corrosion inhibitors for carbon–steel (OL) in an acid medium giving a best performance of 88% in the case of coverings with Pt-porphyrin. Finally, the electrocatalytic activity for the hydrogen and oxygen evolution reactions (HER and OER) of the free-base and Pt-metalated A_3_B porphyrins was evaluated in strong alkaline and acidic electrolyte solutions. The best results were obtained for the electrode modified with the metalated porphyrin, drop-casted on a graphite substrate from an N,N-dimethylformamide solution. In the strong acidic medium, the electrode displayed an HER overpotential of 108 mV, at i = −10 mA/cm^2^ and a Tafel slope value of 205 mV/dec.

## 1. Introduction

Porphyrin derivatives, designed to easily distort their planar geometry, are highly recognised for their capacity to act as sensitive materials in formulations of optical [1,2], fluorescent [3,4], potentiometric [5,6] and electrochemical sensors [7,8]. More structures of diversely substituted porphyrins and metalloporphyrins proved their utility in acting as corrosion inhibitors for carbon–steel devices, working in both saline [9,10,11] and aggressive acid media [12,13]. Recently, as the global energy demand increases, and because hydrogen is considered to be a sustainable choice [14,15], its generation via the electrochemical water splitting method [16,17] involves porphyrins and metalloporphyrins as catalytic materials [18].

Among the many toxic compounds that require monitoring, hydroquinone might be one that produces illness by accumulation.

Hydroquinone (HQ) plays an active part in several processes, such as a redox mediator in photovoltaic cells [19], a reducing agent in black and white photographic developers and in the paper industry [20,21]. HQ is contained in cigarette smoke and is formed during the production of coal tar products [22]. It also has an impact across the biological field so this xenobiotic micropollutant of aquatic media requires continuous monitoring [23].

Exposure to hydroquinone should be well within the recommended limits in the case of skin de-pigmentation/lightening preparations for the treatment of melasma (chloasma) and vitiligo [24,25]. An overdose might generate trouble breathing [26] so patients who use HQ formulations must have regular medical follow-up (every 3 months) irrespective of the concentration they are prescribed. A significant increase in the Raman signal of the bands assigned to hydroquinone was observed in patients with melasma after treatment [27].

For this reason, HQ and its oxidation products have been determined across time using diverse chromatographic [28], electrochemical [29] and photoelectrochemical methods [30].

Several of these methods are based on porphyrins. Hydroquinone [31] was recognised by measuring the fluorescence quantum yields using free-base porphyrin–quinone and zinc porphyrin–quinone dyads. The electron acceptor quinone substituent grafted on the porphyrin ring binds the electron donor hydroquinone to form a 1:1 complex named quinhydrone. 

The porphyrin-based Zr Metal–Organic/β-cyclodextrin on a graphite electrode exhibited remarkable reproducibility, stability, selectivity and electrochemical detection performance for HQ in a very large domain of 1–750 μM and with a limit of detection of 0.07 μM [32]. Another covalent organic framework material, based on a porphyrin, namely 5,10,15,20-tetrakis-(4-aminophenyl)-porphyrin, was prepared for the detection of HQ. Improved sensitivity and stability were obtained and the detection limit was 9.0 × 10^−9^ mol/L for HQ and the linear range was from 1.0 × 10^−7^ mol/L to 1.0 × 10^−4^ mol/L [33].

A photoelectrochemical modern approach [30] for the efficient determination of hydroquinone was developed based on AuNPs functionalised-porphyrin/graphene that was deposited on an ITO electrode and the oxidation of HQ monitored at 0 V under white light illumination, with a wide linear response domain, from 20 to 240 nM, and a detection limit of 4.6 nM.

The corrosion-inhibitory effect of porphyrins can be explained by their strong chemisorption on the surface of metals [34] due to both the planarity of the molecule and the extended conjugation of the π-electron system. Metalloporphyrins containing oxygen atoms in the peripheral meso-substituents, per se, or in different combinations with pseudo-binary oxides [35,36], resins [37] or nylon fibres [38], are capable of uniformly coating steel and protecting it from corrosion, in acidic mediums [34,35,36,37,38,39], salty environments [11] and in sweet corrosive mediums [40]. 

In the third order, the generation of hydrogen by means of the water splitting method employs efficient and diversified catalysts [41,42,43,44,45,46,47,48]; including porphyrin derivatives [49] is in great demand in current research. 

The purpose of this work was to search the limits of exploiting a mixed substituted A_3_B porphyrin, 5-(3-hydroxy-phenyl)-10,15,20-tris-(3-methoxy-phenyl)-porphyrin, and its Pt(II) complex that were synthesised and fully characterised, showing the desired optical and aggregation properties needed for diverse applications. Starting from the knowledge that the design for obtaining porphyrins with the desired non-planar conformation relies on core, peripheral and electronic modifications [50,51], and that a larger reduction in the band gap is noticed in the case of porphyrins grafted with hydroxyphenyl groups in comparison with those substituted with carboxy or nitro groups, [52] we decided to synthesise a hydroxy-substituted porphyrin. In addition, due to the fact that recent reports concluded that the Q(0,0), as well as the Soret band, has maximum intensity in the *meta* isomer and decreases in the series *meta* > *para* > *ortho*, and structural modification with *meta* substitution also increases antibacterial properties [53], the second decision was made to obtain an A_3_B porphyrin with all substituents in a *meta* position. The third guiding thought was that metalloporphyrins adopt various intermediate conformations besides the four main nonplanar conformations, namely: ruffled, saddled, domed and waved [54], and that although the hydroxyl group is generally electron donating, when it is in a *meta* position, it behaves as an electron-withdrawing group. The methoxy groups are also electron withdrawing by the inductive effect of the oxygen atom if they are in the *meta* position. Thus, the electron-withdrawing substituents make the macrocycle more electron deficient, and interaction with electron-donating molecules are favoured. 

The pure compounds were firstly used as sensitive materials for sensitive and selective optical and fluorescence detection of hydroquinone. Secondly, the protective properties against carbon–steel corrosion of the newly obtained porphyrin-base and of the Pt(II) complex were tested in an acid medium. In the third case, as part of the search for new structures possessing electrocatalytic properties for the two half-cell reactions involved in water splitting—the oxygen and hydrogen evolution reactions (OER and HER)—the current work evaluates the catalytic activity of this free-base A_3_B porphyrin and its Pt-metalated counterpart. Because porphyrin molecules have the ability to organise into aggregates through non-covalent interactions between neighbouring macrocycles due to peripheral substituents, as well as through coordinate bonds when a central metal atom is present, they can be used to modify substrates, thus enhancing different properties [49,55,56]. Applying the two porphyrins on nonpolar graphite supports from solutions of solvents having different polarities leads to the formation of diverse arrangements that possess distinct catalytic properties toward the HER and the OER. To the best of our knowledge, neither the sensorial or corrosion inhibition effect, nor the water splitting catalytic activity in strong alkaline and strong acidic environments of these two macrocycles, has been previously reported.

**Scheme 1:**
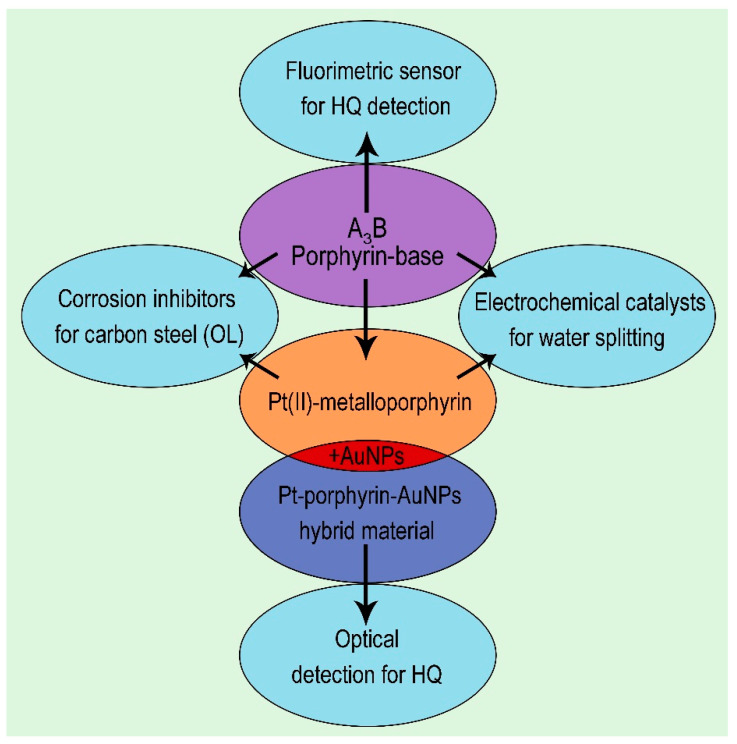
Representation of the three main approaches regarding applications in different fields of the porphyrin-based materials obtained in this work.

In Scheme 1, we present the developments realised in this research.

## 2. Materials and Methods

### 2.1. Materials

PtCl_2_(PhCN)_2_ was produced by Alfa (Haverhill, MA, USA); CH_3_COONa × 3H_2_O, N,N-dimethylformamide (DMF), tetrahydrofuran (THF), dichloromethane (DCM) and potassium hexacyanoferrate(III) were purchased from Sigma Aldrich, (Saint Louis, MO, USA); chloroform, dichloromethane and diethyl ether were provided by Supelco, Merck KGaA, (Darmstadt, Germany). Ethanol was from Honeywell (Charlotte, NC, USA). Chlorobenzene, dimethyl sulfoxide (DMSO), benzonitrile (BN), sulfuric acid 98% potassium nitrate sodium citrate, DL-menthol, KI, calcium gluconate (CaG), lactic acid (LA), sodium acetate (SA) and sodium salicylate (SS) were from Merck (Darmstadt, Germany). HAuCl_4_ × 3H_2_O was supplied by Roth (Karlsruhe, Germany). Acetone, ascorbic acid (AA), NaCl, urea, glucose (Glu) and KCl were provided by Chimreactiv/Reactivul (Bucuresti, Romania). FeCl_3_ was furnished by Fluka Chemie (Buchs, Switzerland). Calcium lactate (CaL) was bought from DH Laboratory Chemicals (Poole, UK). The silica gel was from Scharlab (Barcelona, Spain) (0.04–0.06 mm, 230–400 mesh). The carbon-steel (OL) used in the corrosion tests was type S 185 type (Arcelor Mittal Gipuzkoa, Spain), having the following chemical composition (wt.%): C = 0.15–0.25; Mn = 0.60–0.70; *p* = 0.055; S = 0.055; Fe = 99. Spectroscopic graphite rods (type SW.114 from “Kablo Bratislava”, National Corporation “Electrocarbon Topolcany” Factory, Bratislava, Slovakia) served as conductive support in the electrode-manufacturing process.

### 2.2. Apparatus

A V-650-JASCO spectrometer (Pfungstadt, Germany) was used to record the UV-Vis spectra in 1 cm wide quartz cuvettes. A pH-meter HI 98100 Checker Plus, from Hanna Instruments (Woonsocket, RI, USA) was used to measure the pH values. A Nanosurf^®^ EasyScan 2 Advanced Research AFM microscope (Liestal, Switzerland), equipped with a piezoelectric ceramic cantilever registered the atomic force microscopy (AFM) images of the samples drop-casted on pure silica plates. The emission spectra were registered on a Perkin–Elmer Model LS 55 apparatus (PerkinElmer, Inc./UK Model LS 55, Waltham, MA, USA), using 1 cm path length cells, a scan speed of 100 nm/min, λ_excitation_ = 418 nm, excitation slits of 15 nm, emission slits of 5 nm, at ambient temperature (22−24 °C), without cut-off filters. KBr pellets were used to register FT-IR spectra, on a JASCO 430 FT-IR (Hachioji, Tokyo, Japan) spectrometer, in the range 4000–400 cm^−1^. A 400 MHz Bruker Avance NEO Spectrometer (Rheinsteitten, Germany) equipped with 5 mm four nuclei (1H/13C/19F/29Si) provided the NMR spectra registered in CDCl_3_. The chemical shifts are expressed in (ppm), using as reference tetramethylsilane (TMS).

Scanning electron microscopy (SEM) was performed on a Tescan Vega 3 LMH microscope (Brno, Czech Republic) with an Energy Dispersive X-ray (EDX) detector for the determination of chemical composition. Geometrical evaluation (size and shape), the crystalline structure of nanoparticles and the nanostructure of the samples were investigated by high-resolution transmission electron microscopy (HR-TEM), using a TECNAI F30 G^2^ S-TWIN microscope operated at 300 kV with an Energy Dispersive X-ray Analysis (EDAX) facility. Selected area electron diffraction (SAED) for crystalline structure evaluation was also performed.

For the electrochemical tests of the deposited porphyrins’ thin films on steel, a Voltalab Model PGZ 402 potentiostat (Radiometer Analytical, Copenhagen, Denmark) was used. The calculation of corrosion parameters was performed with VoltaMaster 4 software v.7.09. The setup for the electrochemical measurements comprised an electrochemical cell with three electrodes, i.e., the evaluated sample—either (OL)—for control or porphyrin-modified (by drop-casting) steels disks, a counter electrode (a platinum wire) and a standard reference electrode (Ag/AgCl electrode), connected to a potentionstat (Voltalab Model PGZ 402). All the potentials were referenced to a standard hydrogen electrode (SHE). The potential range scanned during the potentiodynamic polarisation measurements was from −1.3 V to −0.6 V at 1 mV/s scan rate in 0.1 M HCl solution at 23 ± 1 °C. Before polarisation, the OCP—open circuit potential—was monitored for 30 min. The 0.28 cm^2^ constant active surface of the specimens was ensured by mounting the disks in a Teflon body before immersing the electrode into the HCl solution. To calculate the values of corrosion potential (E_corr_), corrosion current density (i_corr_), polarisation resistance (R_p_), corrosion rate (ν_corr_), the anodic Tafel slope (β_a_) and the cathodic Tafel slope (β_c_), VoltaMaster 4 v. 7.09 software was used. The equation published in [57] was applied to determine the value of inhibition efficiency (IE %).

### 2.3. Working Electrodes Preparation

Initially the graphite rods (⌀ = 6 mm) were introduced into polyethylene tubes that became tightly attached to them through a thermal treatment at 180 °C. During the electrochemical experiments, one end of each rod was connected to the potentiostat, while the other end was immersed into the electrolyte solution after its surface had been modified with one of the porphyrins. The modification procedure consisted of several steps: (a) the rod end surface was polished with silicon carbide paper of different grit sizes (800 and 1200) and with felt; (b) the polished surface was washed with double-distilled water, ethanol and acetone; (c) after drying at 40 °C, a volume of 10 µL porphyrin solution was drop-casted on the polished surface; (d) finally, the modified surface was dried at 40 °C for 4 h and at room temperature for 20 h.

The porphyrin solutions, of 3 mM concentration, were obtained by dissolving the two species in organic solvents having different polarities, during a 60-min ultrasonication treatment. The order of decreasing solvent polarity is DMSO > DMF > BN > THF > DCM [58]. Because the solubility of the free-base porphyrin in DMSO was not as good as that of the metalated porphyrin, only Pt-OH-3MeOPP was employed to manufacture modified electrodes using this solvent. 

The resulting modified electrodes were labelled as presented in Table 1.

### 2.4. Electrochemical Experiments to Evaluate the Electrocatalytic Properties for the HER and OER of the Porphyrin-Modified Electrodes

The electrochemical setup chosen to evaluate the electrocatalytic properties for the HER and OER of the porphyrin-modified electrodes consisted in a glass cell and a Voltalab PGZ 402 potentiostat (from Radiometer Analytical) that was connected to an auxiliary electrode (Pt plate with S_geom_ = 0.8 cm^2^), an Ag/AgCl (sat. KCl) reference electrode and to the working electrode (S_geom_ = 0.28 cm^2^). Each of the electrodes labelled in Table 1 was employed as a working electrode and their catalytic activity for the two specified reactions was studied in 1 M KOH and 0.5 M H_2_SO_4_ electrolyte solutions. Polarisation curves, iR-corrected using the Ohmic Drop Comp. option from the Volta Master 4 potentiostat software, were recorded at a scan rate (v) of 5 mV/s—selected in accordance with studies reported in the literature [59,60]. Prior to each HER experiment, the electrolyte solution was deoxygenated by bubbling nitrogen for 30 min. Unless otherwise specified, the electrochemical potential (E) values are represented versus the reversible hydrogen electrode (RHE) using Equation (1) [61], while the current density (i) values refer to the geometric current density. The OER overpotential was obtained with Equation (2) [61] and the HER overpotential with Equation (3).

(1) $$E_{\mathrm{RHE}}={\;E}_{\mathrm{Ag}/\mathrm{AgCl}\left(\mathrm{sat}.\;\mathrm{KCl}\right)}+0.059\times \mathrm{pH}+0.197$$

(2) $$\eta_{O2}=E_{\mathrm{RHE}}-1.23$$

(3) $$\eta_{H2}=\left|E_{\mathrm{RHE}}\right|$$

where: E_RHE_ is the reversible hydrogen electrode potential [V], E_Ag/AgCl(sat. KCl)_ is the potential vs. the Ag/AgCl (sat. KCl) reference electrode [V], η_O2_ is the oxygen evolution overpotential and η_H2_ is the hydrogen evolution overpotential [V].

For the most catalytically active electrode identified in the study, the electroactive surface area (EASA) was estimated using Equation (4)—the Randles–Sevcik equation [62].

(4) $$I_{p}=\left(2.69\times 10^{5}\right)\times n^{3/2}\times A\times D^{1/2}\times C\times v^{1/2}$$

where: I_p_ = the peak current [A]; n = the number of electrons involved in the redox process at T = 298 K; A = the surface area of the working electrode [cm^2^]; D = the diffusion coefficient of the electroactive species [cm^2^/s]; C = the bulk concentration of the electroactive species [M] and v = the scan rate [V/s].

In the case of the ferrocyanide/ferricyanide redox system n = 1 and the reported theoretical value for the diffusion coefficient is 6.7 × 10^−6^ cm^2^/s [62,63].

### 2.5. Method for Obtaining A_3_B Porphyrin Derivatives

#### 2.5.1. Method for Obtaining 5-(3-Hydroxy-phenyl)-10,15,20-tris-(3-methoxy-phenyl)-porphyrin (OH-3MeOPP)

The porphyrin-base, namely: 5-(3-hydroxy-phenyl)-10,15,20-tris-(3-methoxy- phenyl)-porphyrin (OH-3MeOPP), was synthesised by using a multicomponent reaction, involving two different substituted aldehydes, 3-methoxybenzaldehyde and 3-hydroxybenzaldehyde, as it was recently reported in [64]. The mixture of the six obtained compounds was subjected to TLC analysis performed on silica gel 60 F Merck plates and, after elution with chloroform/dichloromethane/diethyl ether (5:5:1, *v*/*v*/*v*), the peak at R_f_ = 0.909 was proven to be the desired OH-3MeOPP A_3_B porphyrin. This was isolated by elution on a silica gel-filled column with chloroform/dichloromethane/diethyl ether (5:5:1, *v*/*v*/*v*) mixture as the first separated product.

The main characteristics of 5-(3-hydroxy-phenyl)-10,15,20-tris-(3-methoxy-phenyl) -porphyrin (OH-3MeOPP) were as follows: violet crystals; yield 13%; m.p. over 320 °C. **^1^H-NMR** (CDCl_3_, 400 MHz), δ, ppm: 8.91–8.89 (m, 8H, **β-pyrrole**), 8.09 (d, 1H, J = 7.2 Hz, **H-4′**), 7.90 (s, 1H, **H-2′**), 7.82 (d, 3H, J = 7.52 Hz, **H-4**), 7.79 (s, 3H, **H-2**), 7.74 (t, 1H, J = 7.94 Hz, **H-5′**), 7.65 (t, 3H, J = 7.64 Hz, **H-5**), 7.54 (d, 1H, J = 7.88 Hz, **H-6′**), 7.34 (dd, 3H, J = 2.08 Hz, **H-6**), 3.99 (s, 9H, -OC**H_3_**), −2.79 (m, 2H, N**H**). **^13^C-NMR** (CDCl_3_, 100 MHz), δ, ppm: 157.98 (**C3**-OCH_3_), 149.36 (**C-3′**), 143.48 (**C-1**), 143.43 (**C-1′**), 132.10 (**C-4′**), 131.41(**C-β-pyrrole**), 129.50 (**C**-C1 *meso* quaternary), 127.94 (**C-2′**), 127.68 (**C-4**), 127.56 (**C-5**), 120.99 (**C-6′**), 120.48 (**C-2**), 119.86 (**C-α**), 113.6 (**C-6**), 55.50 (O**C**H_3_). **UV-Vis**, THF (λ_max_ (log ε)): 417 (5.37); 513 (4.06); 547 (3.63); 590.5 (3.49); 648.5 (3.39). **TLC** (Eluent: chloroform/dichloromethane/diethyl ether (5:5:1, *v*/*v*/*v*)), Rf = 0.909. **FT-IR** (KBr), cm^−1^: 3734 (ν_O-H_); 3314 (ν_N-H_); 2922–2856 (ν_C-H sym and antisym_); 1588 (ν_C=C_); 1462 (ν_C=N_); 1158 (ν_C-O_); 974 (γ_C-H_); 793 (γ_C-Hpyrrole_); 723 (δ_CH3_).

#### 2.5.2. Method for Obtaining Pt(II)-5-(3-Hydroxy-phenyl)-10,15,20-tris-(3-methoxy- phenyl)-porphyrin (Pt-OH-3MeOPP)

**Figure 1:**
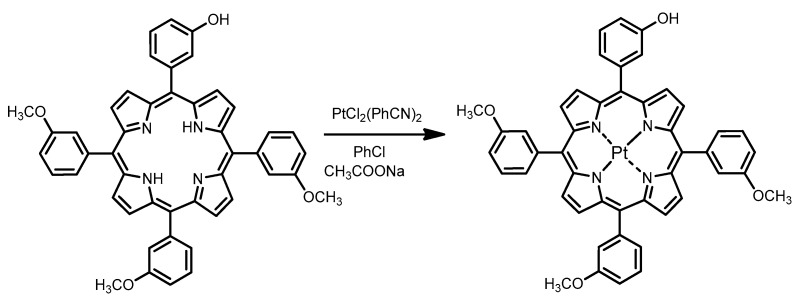
Reaction scheme for obtaining Pt(II)-5-(3-hydroxyphenyl)-10,15,20-tris-(3-methoxyphenyl)porphyrin.

The reaction scheme is presented in Figure 1. A quantity of 0.0854 g (1.18477 × 10^−4^ mmol) of 5-(3-hydroxy-phenyl)-10,15,20-tris-(3-methoxy-phenyl)-porphyrin (OH-3MeOPP) was dissolved in 29 mL chlorobenzene and brought to reflux. A mixture comprising 0.0839 g (1.7716 × 10^−4^ mmol) bis-(benzonitrile)-platinum dichloride (PtCl_2_(PhCN)_2_) and 0.06446 g (4.73908 × 10^−4^ mmol) sodium acetate trihydrate (CH_3_COONa×3H_2_O) suspended in 21 mL chlorobenzene (PhCl) were then added. The refluxing was maintained for 2 h. The reaction mixture was left to cool and then filtered the next day. The filtrate was repeatedly washed with distilled water (4 × 100 mL) in a separatory funnel. The organic extract was then dried over anhydrous Na_2_SO_4_ and next concentrated under vacuum. The precipitate was repeatedly washed with hot water (4 × 25 mL), dried and extracted with THF, to give a yield of 89%.

The main physical–chemical characteristics for Pt(II)-5-(3-hydroxy-phenyl)-10,15,20-tris-(3-methoxy-phenyl)-porphyrin (Pt-OH-3MeOPP) were: orange crystals; yield 89%; m.p. over 320 °C. **^1^H-NMR** (CDCl_3_, 400 MHz), δ, ppm: 9.13 (s, -O**H**), 8.80 (m, 8H, β-pyrrole), 8.02 (d, 1H, J = 7.05 Hz, **H-4′**), 7.89 (s, 1H, **H-2′**), 7.75–7.71 (m, 7H, **H-4**, **H-2**, **H-5′**), 7.62 (t, 3H, J = 7.67 Hz, **H-5**), 7.52 (m, 1H, **H-6′**), 7.31 (dd, 3H, J = 2.26 Hz, **H-6**), 3.97 (s, 9H, OC**H_3_**). **UV-Vis**, THF (λ_max_ (logε)): 400.5 (5.24); 508.5 (4.20); 538 (3.66); 585 (2.84). **FT-IR** (KBr), cm^−1^: 3733 (ν_O-H_); 2923–2856 (ν_C-H sym and antisym_); 1590 (ν_C=C_); 1456 (ν_C=N_); 1162 (ν_C-O_); 789 (γ_C-Hpyrrole_); 704 (δ_CH3_).

## 3. Results and Discussions

### 3.1. Spectroscopic Characterisation

#### 3.1.1. Comparison of OH-3MeOPP and Pt-OH-3MeOPP NMR Spectra

**Figure 2:**
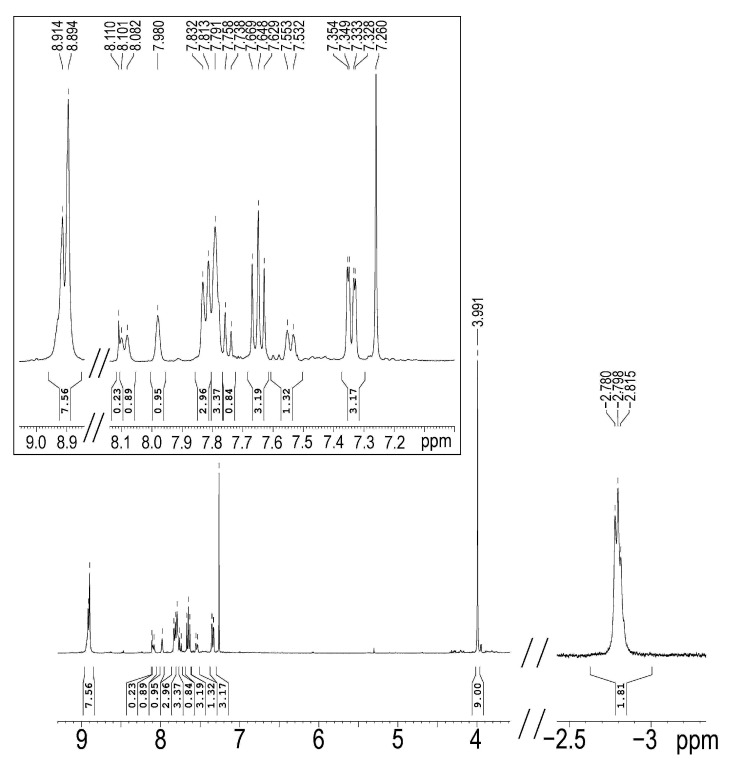
The ^1^H-NMR of (OH-3MeOPP) in CDCl_3_.

The ^1^H-NMR spectrum of OH-3MeOPP (Figure 2) shows, as expected, the signal of the two internal porphyrin protons at −2.79 ppm. This asymmetrical substituted porphyrin shows an important decrease in the current of the cycle leading to the shielding of the two NH protons in the porphyrin core and the corresponding un-shielding of the eight β protons in pyrrole groups. Thus, the β-pyrrolic protons resonate as doublet signal in the interval 8.91–8.89 ppm, while the sixteen aromatic protons gave resonance as multiplet signals in the range 8.10–7.33 ppm. The nine equivalent protons from the methoxy group are identified as singlet at 3.99 ppm.

**Figure 3:**
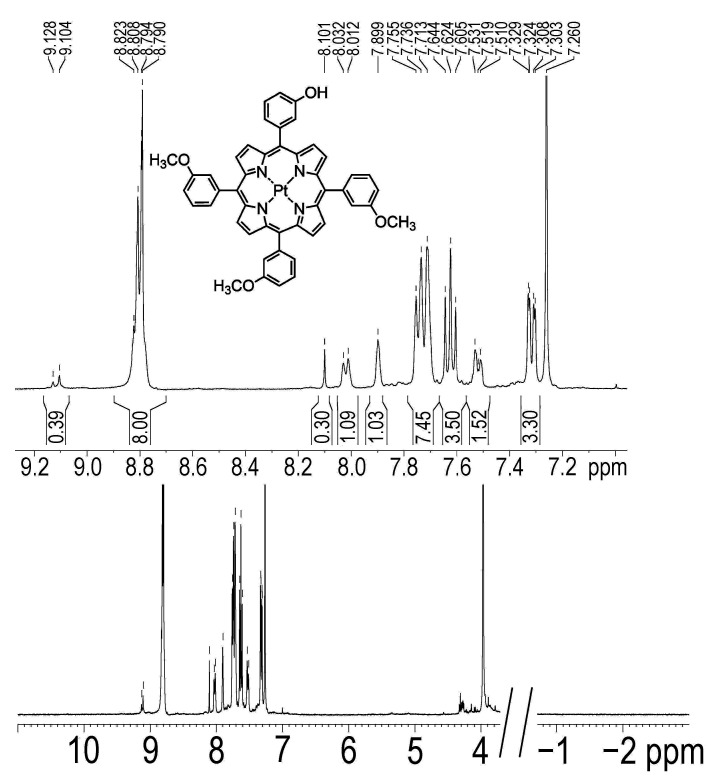
The ^1^H-NMR of Pt-OH-3MeOPP in CDCl_3_.

The ^1^H-NMR spectrum of Pt-OH-3MeOPP (Figure 3) confirms the platinum complexation by a porphyrin ligand showing no signal at −2.79 ppm that is attributed to the core NH group protons in the porphyrin-base. The presence of the metal causes an upfield shift of all the signals from the spectrum.

**Figure 4:**
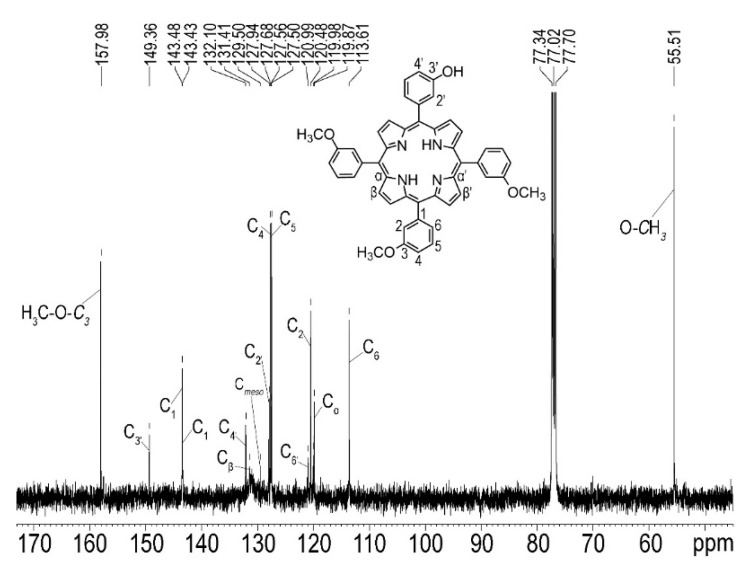
The ^13^C-NMR spectrum of OH-3MeOPP.

The ^13^C NMR spectrum of OH-3MeOPP (Figure 4) shows the signal of the aliphatic-CH_3_ carbon atom at 55.50 ppm. The pyrrolic carbon atom gives a signal at 131.40 ppm and the carbon atoms from the four benzene rings are present in the 149.36–113.60 ppm interval. The aromatic carbon atoms linked to methoxy groups produce a resonance signal at 157.97 ppm. 

#### 3.1.2. Discussion Regarding UV-Vis Spectra of OH-3MeOPP and Pt-OH-3MeOPP

**Figure 5:**
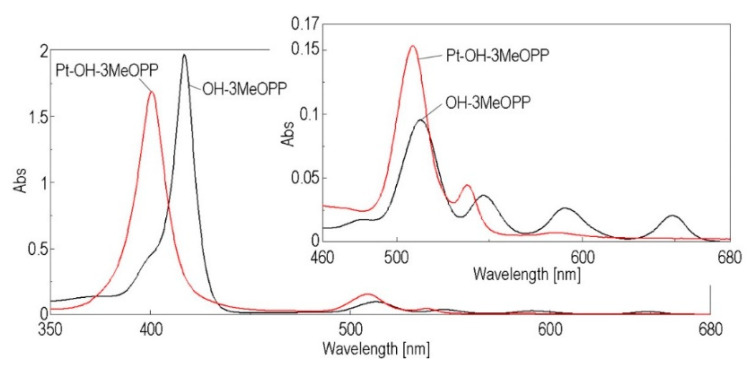
Superposed UV-Vis spectra of OH-3MeOPP (c = 8.2627 × 10^−6^ M) and Pt-OH-3MeOPP (c = 9.629 × 10^−6^ M) registered in THF.

The UV-Vis spectrum of OH-3MeOPP shows *etio* type allure. The absorption spectrum displays the intense Soret band that is accompanied in the visible region by four Q-bands, increasing in intensity in the following order: QI < QII < QIII < QIV, as presented in Figure 5. The Soret band at 417 nm is generated by the transition from a_1u_ (π) − e_g_* (π) and the four Q bands are the result of a_2u_ (π) − e_g_* (π) transitions.

From Figure 5 it can be observed that the metalation of OH-3MeOPP leads to a considerable hypsochromic shift of the Soret band of the Pt-porphyrin derivative in comparison with the porphyrin-base, from 417 nm to 400.5 nm. The reduction in the number of Q bands from four in the porphyrin base to two in the Pt-porphyrin is also noticed, along with their consistent hypsochromic shift, of around 5 nm.

- Effect of protonation Protonation on UV-Vis spectra Spectra of OH-3MeOPP

The effect of protonation on porphyrin bases is notorious for the increasing of the optical properties, both regarding the widening of the absorption bands in the UV-Vis spectrum of OH-3MeOPP and also regarding the hyperchromic benefits. These modifications are discussed in detail in Supplementary Materials and presented in Figure S1.

#### 3.1.3. Comparative Aspects of OH-3MeOPP and Pt-OH-3MeOPP FT-IR Spectra 

A comparison regarding the FT-IR spectra of OH-3MeOPP and the Pt-metalloporphyrin is discussed in Supplementary Materials, Figure S2, emphasising the main differences. 

### 3.2. Combined Microscopic Investigations

Geometrical evaluation (size and shape), crystalline structure of nanoparticles, the nanostructure and the chemical nanocomposition of the samples were investigated by combined methods of electron microscopy: scanning electron microscopy (SEM), transmission electron microscopy (TEM) and high-resolution transmission electron microscopy (HR-TEM), both with the Energy Dispersive X-ray Analysis (EDAX) facility used to characterise the morphology and the type of aggregate architectures of the OH-3MeOPP and Pt-OH-3MeOPP.

**Figure 6:**
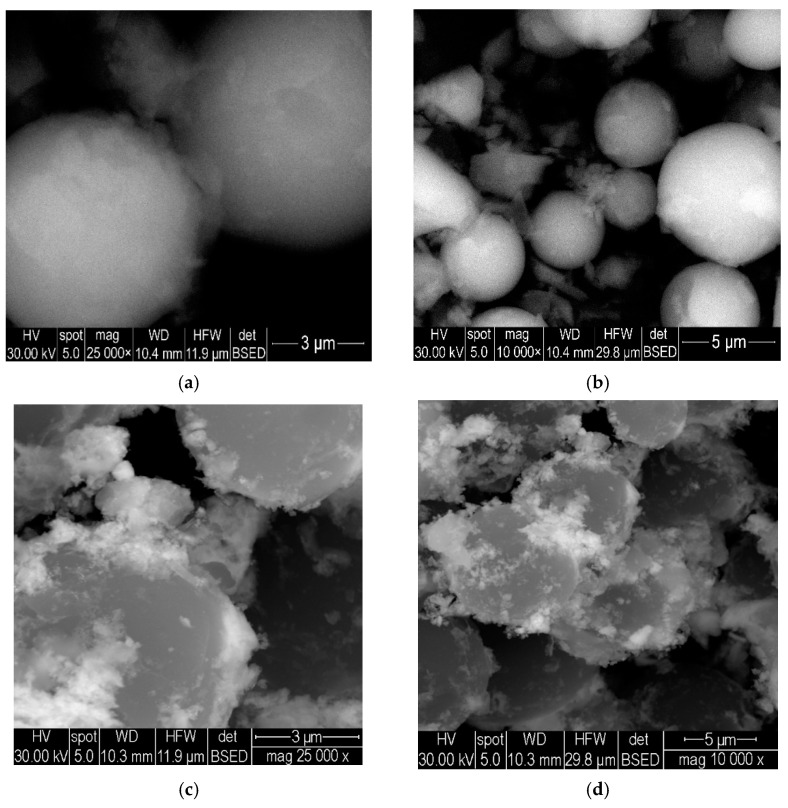

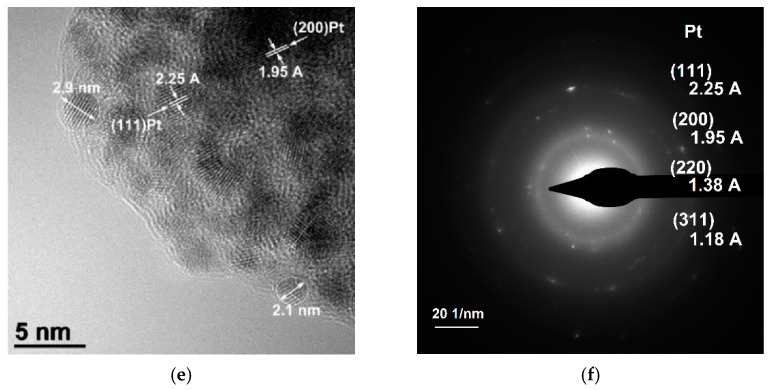
SEM images (backscattered electron images) for large spherical aggregates of OH-3MeOPP (**a**,**b**); SEM (backscattered electron) images with hemisphere assemblies of different dimensions with the interior entirely filled for Pt-OH-3MeOPP (**c**,**d**) and an HR-TEM image (**e**) for Pt-OH-3MeOPP showing the (111) and (200) crystallographic planes of Pt, SAED image revealing the (111), (200), (220), and (311) crystalline planes of the Pt-OH-3MeOPP (**f**).

Figure 6a,b displays the SEM images for large spherical aggregates of OH-3MeOPP. Figure 6c,d shows SEM images of hemisphere assemblies of different dimensions with the interior entirely filled for Pt-OH-3MeOPP. Figure 6e,f presents the HR-TEM image for Pt-OH-3MeOPP showing the (111) and (200) crystallographic planes and the selected area electron diffraction (SAED) image (f) revealing the (111), (200), (220), and (311) crystalline planes of the platinum from Pt-OH-3MeOPP.

**Figure 7:**
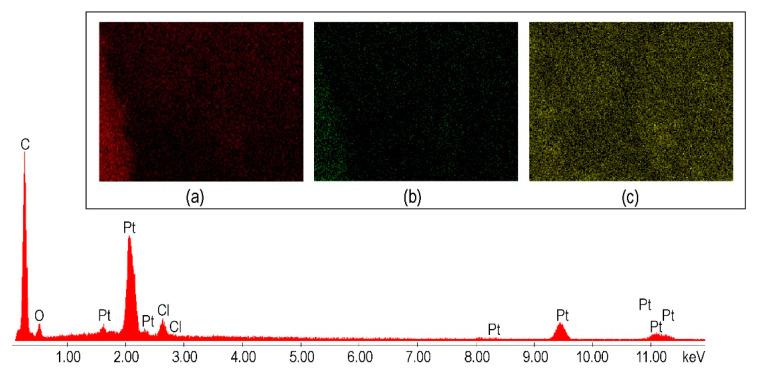
EDX spectrum for Pt-OH-3MeOPP; in detail, map CK (**a**), map OK (**b**) and map PtM (**c**).

Crystalline nanoparticles (Figure 6e) with diameters of almost 2 and 3 nm are visible embedded in an amorphous-type matrix. The parallel fringes in Figure 6e correspond to crystallographic planes with (111) and (200) Miller indices. The diffraction rings in the (SAED) image in Figure 6f are assigned to the Miller families of planes (111), (200), (220) and (311) of the Pt compound. The measured crystallographic parameters of Pt-OH-3MeOPP are: cubic crystal system, Fm-3m space group (No. 225), a(Å) = b(Å) = c(Å) = 3.9030, as indicated by Ref Pattern Platinum 04-001-2680. Figure 7 represents the EDX spectrum for Pt-OH-3MeOPP and the distribution maps for C (a), O (b) and Pt (c). 

Adsorption of benzene and phenol was studied in detail on PtM and PtM_3_ (111) surfaces with excellent results [65], completely justifying our approach to study the interaction of this Pt-porphyrin with HQ.

The goniometric phases for OH-3MeOPP and Pt-OH-3MeOPP, with assignment of Pt signals, are given in Supplementary Materials, Figure S3.

### 3.3. Generation of the Pt-OH-3MeOPP-AuNPs Complex

#### 3.3.1. AuNPs Colloid Preparation

A gold colloidal solution was prepared by an adapted method [66,67] starting with 0.0455 g (0.155 mmol) tetrachloroauric acid trihydrate HAuCl_4_ × 3H_2_O dissolved in 151.06 mL distilled water. The solution was brought to reflux and then 8.75 mL solution of trisodium citrate (c = 1%) was immediately added. The reflux was maintained for another 15 min, when the solution colour changed from yellow to black and, finally, to dark red. A gold colloidal solution having a concentration of c = 9.698 × 10^−4^ M was obtained.

#### 3.3.2. Method for Obtaining the Pt-OH-3MeOPP-AuNPs Complex

**Figure 8:**
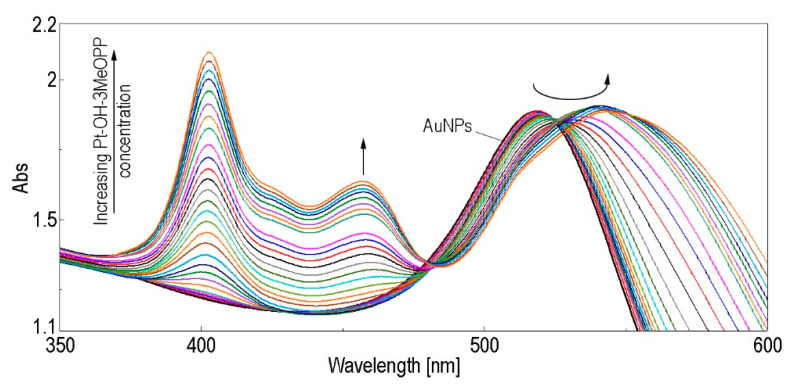
UV-Vis monitoring of the Pt-OH-3MeOPP-AuNPs complex formation in THF/water (1/9, *v*/*v*).

To a quantity of 5 mL of gold colloidal solution (7.758 × 10^−4^ M), different portions of Pt-OH-3MeOPP solution in THF (c = 2.40725 × 10^−5^ M) were added. After each addition, stirring was maintained for 90 s and then the UV-Vis spectrum was recorded (Figure 8).

It can be observed that the Pt-OH-3MeOPP-AuNPs complex has improved optical properties, considerably enlarging the absorption domain in comparison with both the gold plasmon and the Pt-metalloporphyrin. The plasmonic band of the gold colloid is bathochromically shifted, from 519 nm to 544 nm, associated with a hyperchromic effect. A novel peak arises at 457 nm. 

The linear dependence between the intensity of absorption read at the wavelength of the newly formed peak (457 nm) and the Pt-OH-3MeOPP concentration is presented in Supplementary Materials, Figure S4, being important for trace detection of porphyrins.

### 3.4. Detection of HQ Use as Sensitive Material A_3_B Porphyrin Derivatives

#### 3.4.1. UV-Vis Detection of HQ by Acidulated Pt-OH-3MeOPP-AuNPs Complex

**Figure 9:**
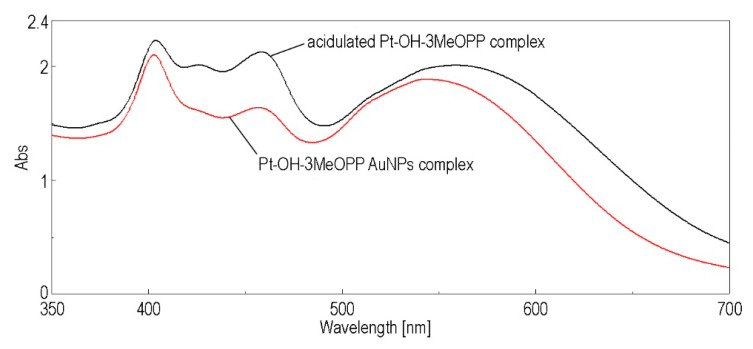
Overlapped UV-Vis spectra of the Pt-OH-3MeOPP-AuNPs complex before and after acidulation.

The complex formed between Pt-OH-3MeOPP and AuNPs that possessed the largest absorption domain (molar ratio AuNPs/Pt-OH-3MeOPP = 13.39/1) was acidulated with HCl (c = 0.5 M) to pH = 2.5, as presented in Figure 9.

As a consequence of acidulation, the absorption domain continues to widen, from 403 nm to 559 nm due to pronounced aggregation in the acid aqueous solution of the porphyrin. Besides, the plasmonic band also enlarges so that the maximum absorption intensity shifts to longer wavelengths, from 543.5 nm to 559 nm, accompanied by an increase in intensity. 

**Figure 10:**
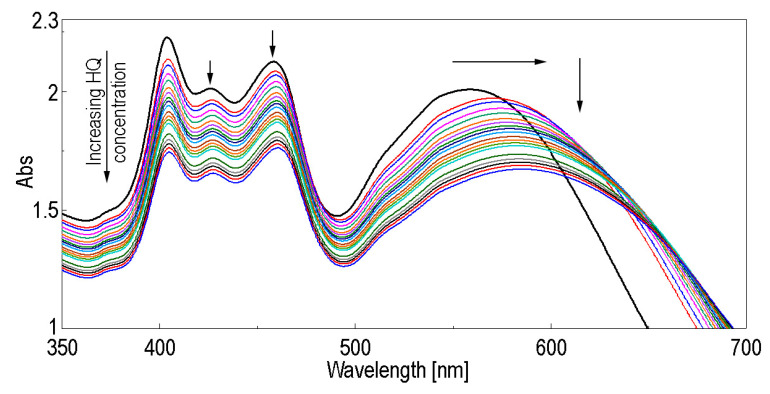
Overlapped UV-Vis spectra after exposure to HQ of the acidulated Pt-OH-3MeOPP-AuNPs complex, in THF/water (1/9, *v*/*v*).

To a quantity of 2.5 mL acidulated complex, portions of 0.01 mL HQ solution in water (c = 1 × 10^−5^ M) were added. After each HQ addition, the mixtures were stirred for 90 s. The overlapped UV-Vis spectra are presented in Figure 10.

**Figure 11:**
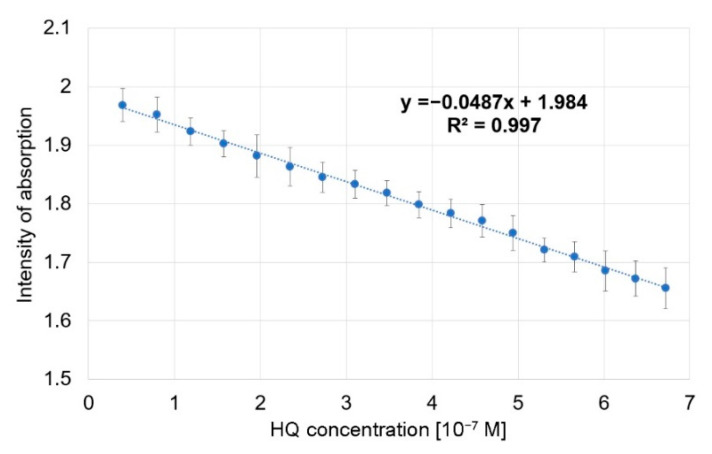
Linear dependence between the intensity of absorption of Pt-OH-3MeOPP-AuNPs complex read at 570 nm and the HQ concentration, in THF/water (1/9, *v*/*v*).

From Figure 10 it can be noticed that the adding of HQ produces both a continuous diminishing of the intensity of absorption of all bands and a bathochromic shift of the plasmonic band, from 559 nm to 585 nm. The calibration curve was obtained after three experiments, calculating the average intensity values. The error bars were established using the standard deviation function in an Excel worksheet (standard deviation ranges from 0.02 to 0.036). The dependence between the intensity of absorption read at 570 nm and the HQ concentration (Figure 11) is linear in the concentration interval 3.98 × 10^−8^ M to 6.71 × 10^−7^ M, which is relevant for the quantification of HQ in blood and urine before it reaches the dangerous human toxicity level [68]. Benzene-induced leukaemia is produced by HQ and other benzene metabolites. Sources of high HQ levels are cigarette smoke (110 to 300 µg per cigarette) and acetaminophen (which is metabolised to HQ and other products) [69].

The limit of detection (LOD) for HQ optical determination is 0.013 µM and was calculated with the formula LOD = 3.3 σ/S, where σ represents the standard deviation of the responses and S is the slope of the calibration curve [70].

- Interfering species study for the UV-Vis detection of HQ

Because the blood and urine of the patients are the main body fluids subjected to HQ analysis, the selected interfering compounds to be tested were: ascorbic acid (AA), urea, DL-menthol, calcium gluconate (CaGlu), lactic acid (LA), glucose (Glu), KCl, sodium acetate (SA), calcium lactate (CaL), FeCl_3_, Sodium salicylate (SS), NaCl and KI. 

A solution of acidulated Pt-OH-3MeOPP-AuNPs complex was prepared, in which the HQ concentration was 2 × 10^−5^ M. The reference sample comprised 3 mL acidulated Pt-OH-3MeOPP-AuNPs in which 0.1 mL distilled water was added, in order to avoid false results due to dilution when measuring the interferences effects. In each of the other samples, 0.1 mL solution of each interfering compound was added, so that the concentration of the interfering species exceeded 100 times the concentration of HQ in the solution.

Table 2 presents average percentage errors for each interfering species. Average percentage errors were calculated according to the following equation: |∆I/I| × 100, where I is the absorption intensity of the reference containing solely HQ and ∆I represents the difference between I and the absorption intensity of each sample containing both HQ and one of the tested interfering analytes.

From Table 2 it can be noticed that the metal salts NaCl and KI introduce errors in the hydroquinone detection. KI is an avoidable interfering compound, as the average percentage error introduced in the detection process is higher than 16%, as expected from our previous experience [71].

- Detection mechanism

**Figure 12:**
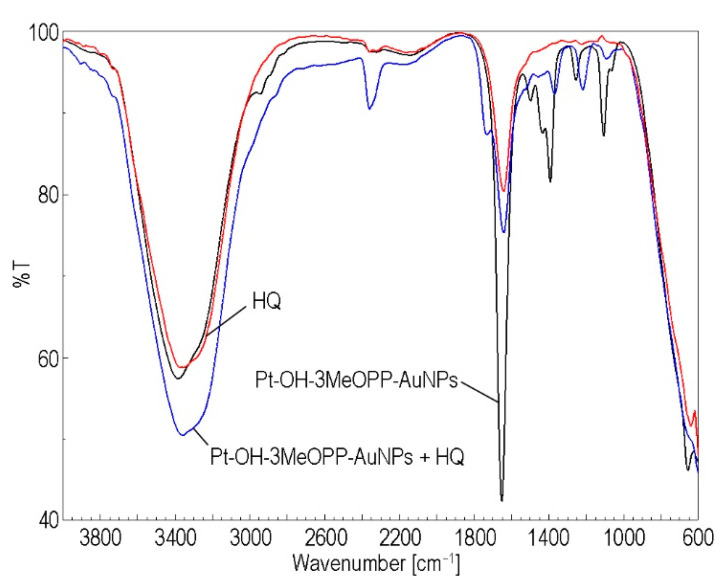
Comparative IR spectra in ATR mode of acidulated Pt-OH-3MeOPP-AuNPs complex, HQ and Pt-OH-3MeOPP-AuNPs complex after HQ detection.

In order to propose a detection mechanism, the superposed FT-IR spectra of Pt-OH-3MeOPP-AuNPs complex, HQ and Pt-OH-3MeOPP-AuNPs complex treated with HQ were compared, as shown in Figure 12. The only significant band that is present after HQ detection is the pronounced shoulder at 1732 nm. This can be assigned to unconjugated carbonyl stretching (C–O stretching) or to the C=O ester band, meaning that hydroquinone generates semiquinone radicals and then 1,4-benzoquinone [72,73] These species are favoured to form OH···O=C weak bonds between the OH-group of functionalised porphyrin and the C=O group of 1,4-benzoquinone, or even a C–O^−^···OH link between the semiquinone and the same OH-group of Pt-OH-3MeOPP [31].

Our next approach was to investigate the two porphyrins as sensitive systems in fluorescence detection of HQ, having as target an increase in the detection range and the selectivity. 

#### 3.4.2. Fluorescence Detection of HQ by OH-3MeOPP in Acid Medium

This investigation was undertaken in an attempt to find a more simplified method for HQ detection, using only the acidified OH-3MeOPP instead of a plasmonic Pt-OH-3MeOPP-AuNPs hybrid material; this was done in the hope of obtaining a better selectivity with respect to KI interference, a lower detection limit and of enlarging the detection domain. These aspects are appropriately discussed in Supplementary Materials, Figures S5−S7. The realised fluorescence sensor did not provide superior sensitivity in comparison with the optical sensor, and only better selectivity in the presence of iodide, as can be seen from Table S1. Thus, when samples from patients with thyroid illness must be analysed, this method offers better precision.

### 3.5. Corrosion Tests

#### 3.5.1. Thin Films Realisation

The porphyrins were deposited in three successive layers from concentrated solutions in THF (c = 1 × 10^−3^ M) by the drop-casting method, on polished steel (OL) disks, having a diameter of 10 mm and a thickness of 2 mm. The thickness of the porphyrin layers deposited by drop-casting were around 120 µm.

#### 3.5.2. Electrochemical Measurements

**Figure 13:**
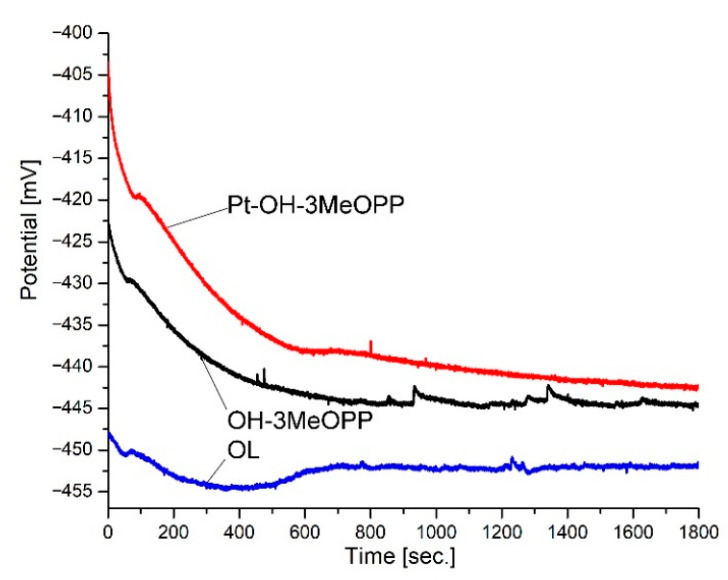
The OCP measurements of the OL and the two covered electrodes after 30 min immersion in 0.1 M HCl.

The OCP measurements (Figure 13) showed that regardless of coating, all electrodes stabilise around 1000 s. Both in the case of bare porphyrin, OH-3MeOPP, and of its Pt-derivative, Pt-OH-3MeOPP, a time of exposure of 1800 s leads to a shift of the free potential towards more negative values. The two porphyrin-modified electrodes have positivated free potential values in comparison with the OL control electrode.

**Figure 14:**
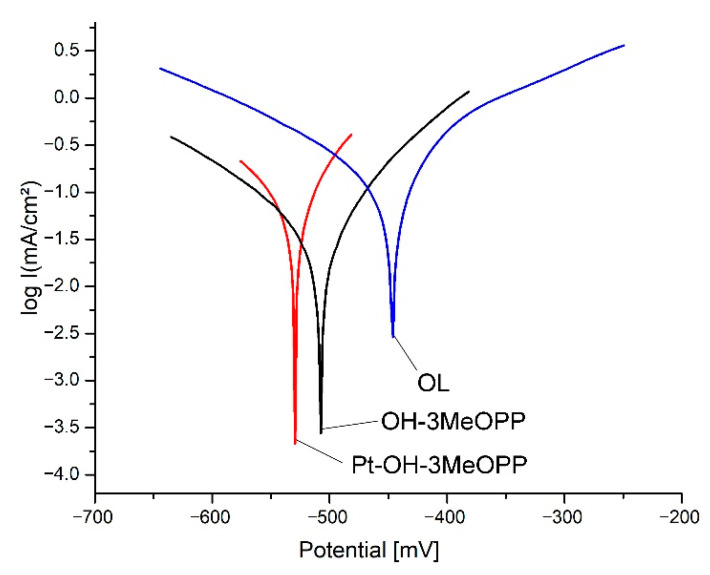
Tafel polarisation curves recorded in 0.1 M HCl for the OL and protected steel electrodes.

The Tafel plots of the investigated OL electrodes recorded after 30 min OCP in 0.1 M HCl solution are represented in Figure 14. The Tafel slopes were determined in anodic and cathodic regions, before and after reaching the corrosion potential (U). The Tafel parameters, which were calculated using VoltaMaster 4 v. 7.09 software, are presented in Table 3. 

The corrosion potential (E_corr_) of the OL control electrode is −446.67 mV and the corrosion current density (i_corr_) has the highest value of 0.1922 mA/cm^2^. The porphyrin thin films drop-casted in three layers on the covered steel electrodes show five to eight times lower corrosion current densities in comparison with the OL disk. The same tendency is noticed regarding the rate of corrosion.

As expected, the polarisation resistance (R_p_) significantly increases both in the case of the OH-3MeOPP covering layer and in the case of the Pt-OH-3MeOPP protective layer from 1.45 to 3.24-fold in comparison with bare OL.

The highest inhibition efficiency of 88.03% resulted for the OL protected by Pt-OH-3MeOPP. This result is sustained by analysing the data presented in Table 3, before and after the corrosion tests, where Pt-OH-3MeOPP provided the smallest differences between Sa, Sq and Sy, before and after tests, proving that the layer is stable and offers protection in the acid medium.

Taking into consideration that the difference of potential between the anodic and cathodic regions decreases from OL > OH-3MeOPP > Pt-OH-3MeOPP (because the anodic slope β_a_ decreases at a slower rate than the cathodic slope β_c_ increases), a lower corrosion rate is expected when using the two porphyrins as corrosion inhibitors, as presented in Table 3 [74].

It can be observed from Table 3 that Pt-OH-3MeOPP offers better protection against corrosion of steel compared with the porphyrin-base OH-3MeOPP, based on the better covering strength of the Pt-porphyrin to the OL surface, as already reported for other Co-, Zn- and Ni- porphyrins [37] due to the superior self-assembling capacity (both J- and H-type aggregation).

#### 3.5.3. AFM Investigation of Covered Steel Electrodes with Two Porphyrin Derivatives

**Figure 15:**
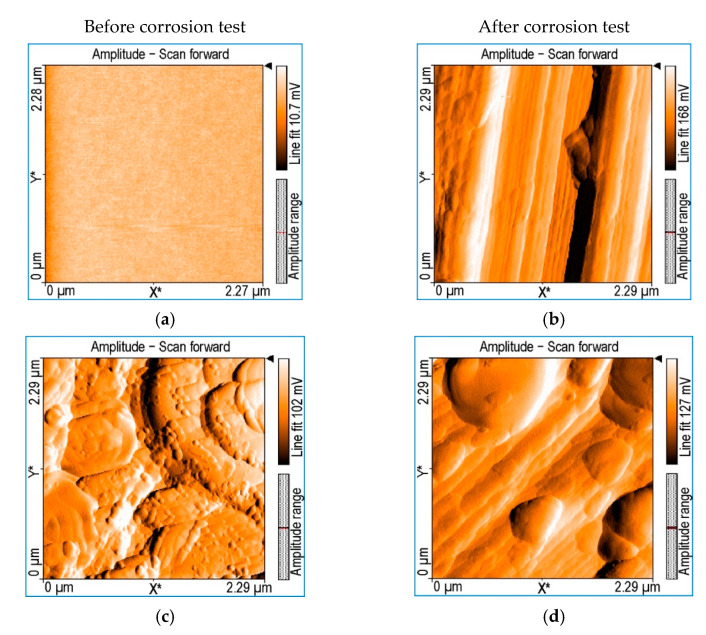

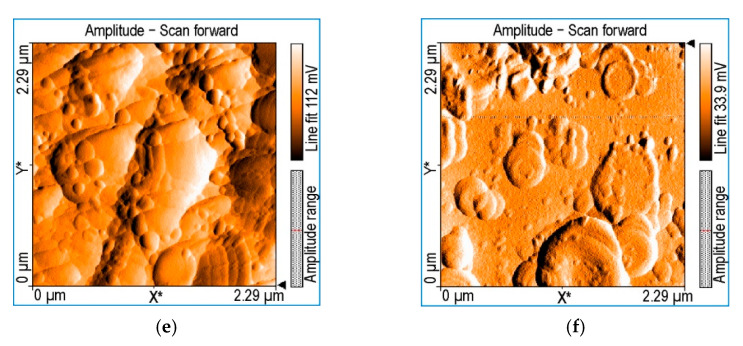
The 2D AFM images before and after exposure to acidic medium of: bare OL (**a**,**b**), carbon-steel electrode covered with OH-3MeOPP (**c**,**d**) and carbon-steel electrode covered with Pt-OH-3MeOPP (**e**,**f**).

The AFM investigation of carbon–steel electrodes covered with OH-3MeOPP porphyrin-base and its metalloporphyrin, Pt-OH-3MeOPP, was performed in non-contact mode before and after exposure to 0.1 M HCl solution used as a corrosive agent, and is presented in Figure 15.

The unprotected steel electrode (Figure 15a,b) that, before exposure to acid, presents a smooth and flat surface is transformed into a ribbed surface with deep unevennness and roughness. The level difference, as measured by Sa and Sq roughness, is around 40 nm in both cases. As expected, this is the highest corrosion effect. 

Regarding the porphyrin-base OH-3MeOPP, the initial covering presents aggregated circular or large pellet-type architectures, based on small triangular building bricks (Figure 15c). After performing the corrosion test in an acid medium (Figure 15d), the surface has a simplified row-type aspect, the triangular brick-shapes have higher dimensions and an uniaxial orientation is noticed. We presume that hydrogen bonding is accountable for these head-to-tail oriented arrangements, because both hydrogen bond acceptor and donor functionalities are present in the OH-3MeOPP structure [75]. The level difference before and after performing the corrosion test, as measured by Sa and Sq roughness, is around 10 nm and 12 nm, respectively—a significantly diminished value in comparison with the unprotected steel electrode (meaning approximately four times lower).

The best result, producing the best barrier against corrosion was given by Pt-OH-3MeOPP, which led to a mixture of triangular and round aggregates on the steel surface before exposure to the corrosive medium (Figure 15e), but completely reorganises after interaction with acid. After exposure to the corrosive acid medium, the surface of the steel protected by the thin layer of Pt-OH-3MeOPP (Figure 15f) is covered with aggregates of overlayed disks. The level difference measured by Sa and Sq roughness, before and after performing the corrosion test, is in this case around 6 nm, that is the lowest value compared with bare OL (Table 4). 

### 3.6. Main Results Regarding the OER and HER Electrocatalytic Activity of the Two Investigated Porphyrin Derivatives, Obtained in Strong Alkaline and Acidic Electrolyte Solutions

#### 3.6.1. Polarisation Curves in Alkaline Medium

**Figure 16:**
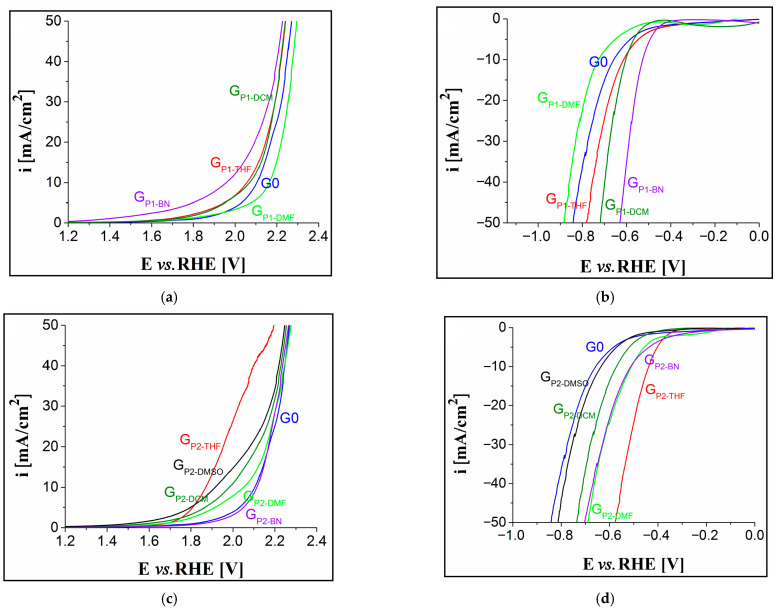
Polarisation curves recorded in 1M KOH solution on the unmodified and the porphyrin- modified electrodes. Anodic curves obtained for the OH-3MeOPP-modified electrodes (**a**). Cathodic curves obtained for the OH-3MeOPP-modified electrodes (**b**). Anodic curves recorded on the Pt-OH-3MeOPP-modified electrodes (**c**). Cathodic curves recorded on the Pt-OH-3MeOPP-modified electrodes (**d**).

The OER and HER polarisation curves recorded in 1 M KOH solution on the electrodes modified with the free-base porphyrin are presented in Figure 16a,b. The G_P1-BN_ electrode was the most catalytically active for both reactions, an observation indicating that OH-3MeOPP could behave as a bifunctional catalyst when drop-casted from BN. Throughout the investigations, the OER and HER overpotential values are specified at i = 10 mA/cm^2^ and i = −10 mA/cm^2^, respectively—in agreement with studies reported in the literature [76,77,78]. Thus, for the G_P1-BN_ electrode, η_O2_ = 0.73 V and η_H2_ = 0.53 V.

The Pt-metalloporphyrin was studied as the catalyst for the HER and OER under the same alkaline conditions as the free-base counterpart. It is already known that the oxygen reduction reaction (ORR) performance of the Pt-based catalysts, especially involving half-sphere structures (as reported in our study in Figure 6d), are favoured by the (111) facet [79].

The anodic curves recorded in the 1M KOH solution are presented in Figure 16c. It can be seen that G_P2-THF_ was more catalytically active than the other modified electrodes (η_O2_ = 0.64 V, at i = 10 mA/cm^2^). The same modified electrode, in the same electrolyte solution, was also evidenced as having the highest HER activity (Figure 16d), which indicates that the metalloporphyrin could act as a bifunctional catalyst when applied from THF. In this medium it displayed a η_H2_ value of 0.437 V, at i = −10 mA/cm^2^.

#### 3.6.2. Polarisation Curves in Acidic Medium

**Figure 17:**
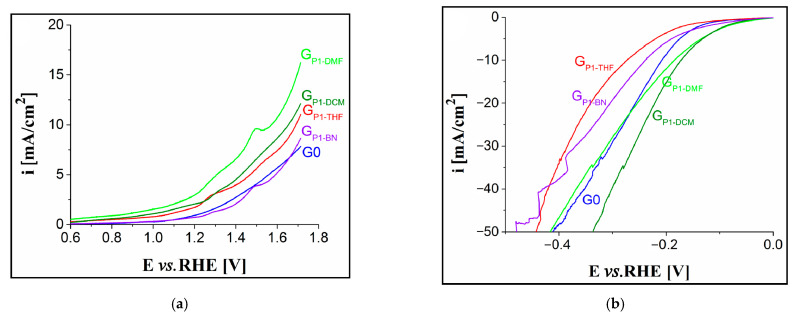

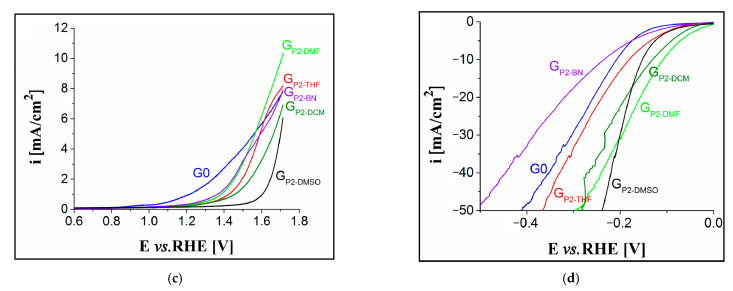
Polarisation curves recorded in 0.5M H_2_SO_4_ solution on the unmodified and the porphyrin-modified electrodes. Anodic curves obtained for the OH-3MeOPP-modified electrodes (**a**). Cathodic curves obtained for the OH-3MeOPP-modified electrodes (**b**). Anodic curves recorded on the Pt-OH-3MeOPP-modified electrodes (**c**). Cathodic curves recorded on the Pt-OH-3MeOPP-modified electrodes (**d**).

Figure 17a,b show the anodic and cathodic polarisation curves obtained for the electrodes modified with the free-base porphyrin in a strong acidic medium. All anodic voltammograms recorded on the modified electrodes display two oxidation signals that are not present on the curve traced using the unmodified electrode. The first signal can be attributed to the formation of the porphyrin cation radical and the second to the formation of the porphyrin dication [80]. In terms of the OER activity of the studied electrodes, G_P1-DMF_ displayed the lowest overpotential value (at i = 10 mA/cm^2^) of 0.34 V. The voltammogram obtained for this electrode shows the oxidation peak attributable to the porphyrin dication at E = 1.5 V. The signal is no longer present at the potential corresponding to i = 10 mA/cm^2^, which means that it does not influence the η_O2_ value at this current density. The HER experiments performed on the modified electrodes revealed G_P1-DCM_ as the most electrocatalytically active. It displayed a η_H2_ value of 0.171 V, at i = −10 mA/cm^2^.

The OER and HER experimental results obtained for the Pt-OH-3MeOPP-modified electrodes, in a strong acidic medium, are presented in Figure 17c,d, respectively. The metalloporphyrin-based electrodes showed poor catalytic activity for the OER, with the lowest η_O2_ value (of 0.47 V) calculated for G_P2-DMF_. Lastly, at i = −10 mA/cm^2^, the same electrode displayed an HER overpotential value of 0.108 V—the lowest among the investigated specimens.

#### 3.6.3. Further Electrochemical Investigations on the G_P2-DMF_ Electrode

**Figure 18:**
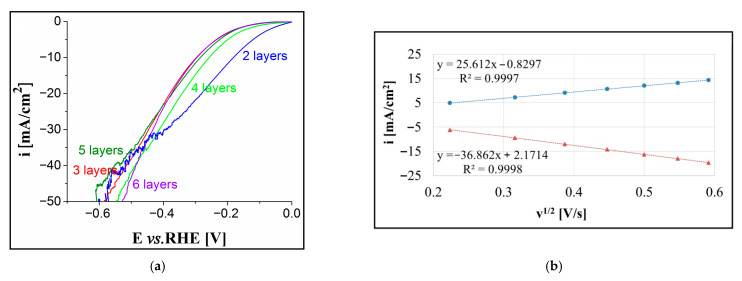

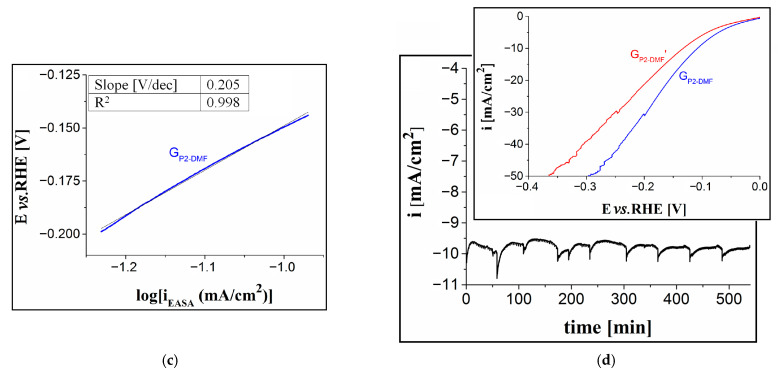
Cathodic polarisation curves recorded in 0.5 M H_2_SO_4_ solution, on electrodes modified with 2 to 6 layers of Pt-OH-3MeOPP drop-casted from DMF (**a**). The plot of the anodic and cathodic current densities vs. the square root of scan rate for the G_P2-DMF_ modified electrode (**b**). Tafel plot obtained for the G_P2-DMF_ electrode in 0.5 M H_2_SO_4_ solution (**c**). i–t curve recorded on the G_P2-DMF_ electrode in 0.5 M H_2_SO_4_ solution and inset showing the cathodic polarisation curves obtained for the same electrode, before and after the stability test, denoted G_P2-DMF_ and G_P2-DMF_’, respectively (**d**).

Because of the low η_H2_ value obtained for G_P2-DMF_, this electrode was selected for further studies. In one such investigation, several electrodes were prepared by applying two to six layers of the metalloporphyrin, from DMF solution, on the graphite supports. The cathodic polarisation curves obtained for these electrodes in a strong acidic medium are presented in Figure 18a. By comparing the results with the voltammogram recorded for the G_P2-DMF_ (Figure 17d), it can be concluded that this electrode, manufactured by drop-casting one layer of Pt-porphyrin, displayed the highest HER catalytic activity. Although it is expected for a thicker film to provide a higher active surface area and thus a faster HER kinetics, usually a thicker and compact film that is neither sufficiently porous nor permeable does not determine higher HER activity [81]. The electroactive surface area (EASA) value of G_P2-DMF_ was estimated using the previously specified Randles–Sevcik equation, together with cyclic voltammetry data from cycles obtained for the electrode in 1 M KNO_3_ electrolyte solution, in the absence and in the presence of 4 mM K_3_[Fe(CN)_6_], at different scan rates (v = 50, 100, 150, 200, 250, 300 and 350 mV/s). The obtained value of 0.51 cm^2^ is almost twice that of the geometric area, and the value of the diffusion coefficient of the electroactive species—calculated with the same equation—was found to be 2.25 × 10^−5^ cm^2^/s. The cyclic voltammetry data were also used to represent the plot of the anodic and cathodic peak current densities vs. the square root of the scan rate (Figure 18b). As the absolute values of the peak current densities increased, so did the scan rate. This relationship of direct proportionality is indicative of a diffusion-controlled electron-transfer process [82], excluding a surface-controlled process for this redox system [83]. Besides, the i_a_/i_c_ ratio is far from the value of 1, meaning that a quasi-reversible process is occurring [84].

- Electrochemical Study of HER Kinetics

The HER kinetics at the interface between the G_P2-DMF_ and the 0.5 M H_2_SO_4_ electrolyte solution were also studied and the Tafel plot, obtained after the current density was normalised to the estimated electroactive surface area (EASA) value (i_EASA_), is shown in Figure 18c. The Tafel slope was determined with the Tafel equation [85], and its value is 0.205 V/dec. Finally, the electrochemical stability of the G_P2-DMF_ electrode was evaluated by chronoamperometry, recording the i-time curve presented in Figure 18d, at the potential value corresponding to i = −10 mA/cm^2^. During the experiment, H_2_ bubbles formed and accumulated on the electrode surface and were subsequently released from it. These phenomena are likely responsible for the pattern observed on the chronoamperogram. The inset from Figure 18d shows the polarisation curves recorded on the G_P2-DMF_ electrode before and after the stability test. It can be seen that the experiment led to an increase in the HER overpotential. At i = −10 mA/cm^2^, η_H2_ increased by 24 mV. This result underlines the stability limitations of the Pt-porphyrin-modified electrode. 

**Figure 19:**
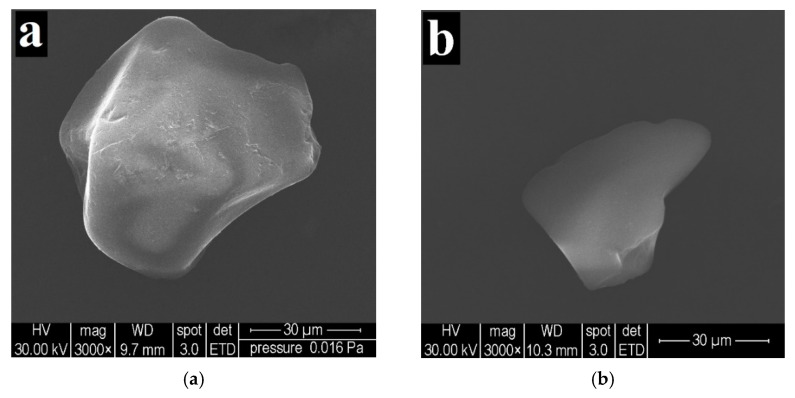
SEM micrographs recorded on the G_P2-DMF_ electrode before (**a**) and after (**b**) the stability test.

In order to verify whether the chronoamperometric experiment affected the morphology of the metalloporphyrin aggregates formed on the graphite surface as a result of the G_P2-DMF_ electrode manufacturing process, SEM analysis of the specimen before and after the stability test was performed, and the scanned images are presented in Figure 19. The results show that the Pt-porphyrin-based arrangements had the same types of shapes after the investigation as they did before the test. Thus, it can be concluded that the experiment did not have a significant impact on the morphology and self-organisation of the metalloporphyrin aggregates.

- Considerations regarding HER catalytic behaviour

A few comments regarding the features influencing the HER catalytic behaviour of the G_P2-DMF_ electrode can be formulated. 

There are two pathways that, through proton reduction on catalytically active sites, lead to the generation of hydrogen during water electrolysis with a catalyst-modified electrode [86]. In both cases, a hydride species results from the reduction of a proton. Subsequently, one of the pathways involves the reaction between two such species in order for the H_2_ molecule to be generated, while, according to the other pathway, the molecule is formed by the reaction between a hydride species and an H^+^-e^−^ couple. 

Concerning metalloporphyrin catalysts, the scientific literature contains multiple studies outlining the fact that the metal atom situated at the centre of the porphyrin macrocycle, in our case the Pt atom of Pt-OH-3MeOPP, acts as the catalytically active site during the HER [87]. This type of action is favoured due to the fact that the overall porphyrin ring exhibits a slight distortion towards a concave shape [88]. 

Regarding the Pt-OH-3MeOPP-modified electrode, on the cathodic polarisation curves no peak appears that can be assigned for the reduction of Pt^2+^ to Pt^0^. This observation is in agreement with other reported results in which, in similar conditions but using Zn(II), Cu(II) and Ni(II) 5,10,15,20-tetrakis-(4-fluoro-2,6-dimethyl-phenyl)-porphyrin, there was no evidence of demetalated porphyrin [89,90]. 

There is another way in which the central metal of the investigated A_3_B metalloporphyrin contributes to the catalytic behaviour of G_P2-DMF_. The charge transfer effects that occur between the porphyrin molecules and the conductive substrate affect the HER catalytic performance of the electrode [91]. In terms of atomic electronegativity, both Pt and N are more electronegative than the C atoms of the graphite support, and this character influences the electron transfer at the metalloporphyrin/graphite interface by making it evolve from the substrate to the Pt-OH-3MeOPP, thus inducing positive charges on the graphite surface. 

With respect to the charge transfer between neighbouring porphyrin molecules, it partly depends on the non-covalent interactions among the peripheral substituents. For example, hydrogen bonds are expected to be formed by the hydroxyphenyl groups during the drying stage of the electrode manufacturing process. 

Furthermore, once the electrode is immersed into the electrolyte solution, hydrogen bonds between the substituents of neighbouring molecules can result through water molecules. Both the hydroxyphenyl and the methoxyphenyl substituents (via the ether oxygen atom) could form this kind of bonding [91]. 

Lastly, it should be pointed out that a high EASA value indicates the presence of inhomogeneities on the electrode surface—such as boundaries and defects—that serve to generate more catalytically active sites [92,93]. As previously stated, the EASA of the G_P2-DMF_ electrode is almost twice that of its geometric area. It is worth mentioning that a different active centre participates in HER, function of the pH conditions [94]. On the other hand, the choice of a metal coordinated in the porphyrin core could be used to optimise a property such as molecular adsorption, modifying the photocatalytic process that would require little or no bias voltage to function [95].

The η_H2_ value of 108 mV, obtained for the G_P2-DMF_ electrode, at i = −10 mA/cm^2^, in 0.5 M H_2_SO_4_, is comparable with the η_H2_ values of 84 mV and 73 mV, corresponding to the same current density and observed in the same strong acidic medium, but obtained for a glassy carbon electrode modified with drop-casted 20% Pt/Vulcan carbon and for a polycrystalline Pt electrode [96]. The Tafel slopes reported for the two electrodes are, however, much smaller than that determined for G_P2-DMF_. The results obtained using the Pt-OH-3MeOPP-modified electrode evidence the potential of this macrocycle as an HER catalyst, and further studies—involving a different electrode manufacturing method—could lead to a more performant specimen.

## 4. Conclusions

The aim of this work was to search for the multifunctionality of suitable substituted porphyrin derivatives and to prove their diversified applications in the formulation of sensors, in protecting layers for corrosion inhibition and in electrocatalytic processes of water splitting. A mixed substituted A_3_B porphyrin, 5-(3-hydroxy-phenyl)-10,15,20-tris-(3-methoxy-phenyl)-porphyrin and its Pt(II)—complex were synthesised, purified and fully characterised by TLC, ^1^H-NMR, ^13^C-NMR, fluorescence, UV-Vis, FT-IR, AFM, TEM, and SEM with EDX microscopy providing evidence of the desired optical and aggregation properties needed for several applications, both in organic solvents and in acidic media. The pure compounds were used firstly as sensitive materials for sensitive and selective optical and fluorescence detection of hydroquinone. The best results, both regarding the detection range and the limit of detection, were obtained by using a plasmonic complex formed between AuNPs and Pt-OH-3MeOPP as sensitive material, successfully detecting HQ from 0.039 µM to 6.71 µM with a detection limit of 0.013 µM. A comparison of these results with those obtained from other sensor devices in recent years is provided in Table 5.

Secondly, the corrosion-inhibiting properties of the two obtained porphyrins, deposited as thin layers on carbon–steel electrodes, were electrochemically investigated in an aggressive acid medium. The best protection against corrosion (IE = 88%) was also obtained when using the metalloporphyrin Pt-OH-3MeOPP.

In the third case, as part of the selection for new structures possessing electrocatalytic properties for the two half-cell reactions involved in water splitting (OER and HER), the free-base A_3_B porphyrin and its Pt-metalated counterpart were evaluated in strong alkaline and acidic electrolyte solutions. The best results were obtained for the electrode modified with the metalated porphyrin, drop-casted on a graphite substrate from N,N-dimethylformamide solution. In a strong acidic medium, the electrode displayed an HER overpotential of 108 mV, at i = −10 mA/cm^2^, and a Tafel slope value of 205 mV/dec, representing promising results.

In all cases, the detection/inhibition or electrocatalytic mechanism was mainly based on the capacity of this A_3_B tetrapyrrolic structure to form intermolecular hydrogen bonds with HQ and with water molecules, or to create adherent protective layers by self-aggregation in the case of steel corrosion. 

## Figures and Tables

**Table 1:** The labels used to identify the electrodes studied in the electrocatalytic experiments.

Electrode Label	Porphyrin	Solvent
G0	-	-
G_P1-DMF_	OH-3MeOPP	DMF
G_P1-BN_	OH-3MeOPP	BN
G_P1-THF_	OH-3MeOPP	THF
G_P1-DCM_	OH-3MeOPP	DCM
G_P2-DMSO_	Pt-OH-3MeOPP	DMSO
G_P2-DMF_	Pt-OH-3MeOPP	DMF
G_P2-BN_	Pt-OH-3MeOPP	BN
G_P2-THF_	Pt-OH-3MeOPP	THF
G_P2-DCM_	Pt-OH-3MeOPP	DCM

**Table 2:** List of tested interfering analytes and average percentage errors for HQ optical detection.

Interfering Analytes	Average Percentage Error |∆I/I| × 100 [%]
Ref = HQ	0
AA	0.04296
Urea	0.15035
DL-menthol	0.20724
CaGlu	0.27981
LA	0.35701
Glu	0.56774
KCl	0.63914
SA	0.80343
CaL	1.36652
FeCl_3_	2.23903
SS	2.29244
NaCl	3.38264
KI	16.5643

**Table 3:** The Tafel parameters calculated for the electrodes immersed in 0.1M HCl medium for 30 min.

Electrode	E (I = 0) (mV)	R_p_ (Ω cm^2^)	i_corr_ (mA/cm^2^)	β_a_ (mV)	β_c_ (mV)	ν_corr_ (mm/Y)	IE (%)
OL	−446.67	168.55	0.1922	149.6	−191.7	2.24	-
OH-3MeOPP	−508.23	245.10	0.0430	-	−131.9	0.50	77.63
Pt-OH-3MeOPP	−528.40	546.58	0.0230	29.7	−48.3	0.26	88.03

**Table 4:** Particle dimensions, electrode surface and nano-roughness determined by atomic force microscopy.

Sample	Area (pm^2^)	S_a_ before/after (nm)	S_q_ before/after (nm)	Particles Dimension before/after (nm)
OL	5.305	0.21/40.31	0.32/44.03	-
OH-3MeOPP		16.53/27.26	22.49/35.43	76.1/97.5
Pt-OH-3MeOPP		2.73/8.46	3.74/11.25	18.6/25.8

**Table 5:** Comparison of the advantages, disadvantages, detection range and limit of detection of hydroquinone, using several types of sensing materials.

Method of Detection	Sensitive Material	Limit of Detection [μM]	Detection Range [μM]	Advantages	Disadvantages	Ref.
Electro- chemical	active pencil lead covered with β -cyclodextrin and tera-kis(4-carboxyphenyl) porphyrin ligands with zirconium	0.07	1−750	- wide detection range - stability - selectivity	- tested only in natural water	[32]
	poly (N-vinylcapro- lactam); rGO@Au; monoclinic bismuth metavanadate	0.6	2.0−30.0 and 30.0−152.0	- bio- compatibility - chemical stability	- temperature sensitive - high LOD	[97]
	acetylene black and gold nanoparticle composite modified glassy carbon electrodes	1	2–500	- selectivity - fast response	- tested only for tap water - high LOD	[98]
	carbon screen-printed electrodes pre-treated in 0.5 mol/L sulphuric acid solution	0.218	0.5−10.0	- simple - low cost	- few interference species studied	[99]
	carboxylic acid-functionalised graphene-modified glassy carbon electrode	0.04	0.1–40.0	- accuracy - fast response - selectivity	-	[100]
	direct green 6 decorated carbon paste electrode	0.11	10–45	- stability - adapted for tap water testing	- narrow detection range, - only few interferences studied	[101]
	glassy carbon electrode modified with cobalt oxide nanorod	0.2	5–200	- large detection range	- no organic compounds present in body fluids studied as interference species	[102]
Electro- catalytic	covalent organic framework based on 5,10,15,20-tetrakis-(4-amino-phenyl)-porphyrin	0.009	0.1−100	- good biocompatibility - high sensitivity and stability	- interference species with low concentrations were tested	[33]
Photo– electrochemical	5-[*p*-(3-Mercapto- propyl-oxy)-phenyl]-10,15,20- triphenyl–porphyrin/AuNPs/graphene	0.0046	0.020−0.24	- sensitivity - stability - low LOD	- no organic compounds present in body fluids studied as interference species	[30]
UV-Vis detection	acidulated Pt-OH-3MeOPP-AuNPs complex	0.013	0.039–6.71	- highly selective - fast response - low LOD -suitable for medical testing including diabetes patients	- narrow detection range	This work
Fluorescence	OH-3MeOPP in acid medium	0.022	0.065–6.35	-highly selective -fast and simple method -high accuracy for testing thyroid illness patients	-narrow detection range	This work

## Data Availability

The data presented in this study are available on request from the corresponding author.

## Supplementary Materials

The following supporting information can be downloaded at: https://www.mdpi.com/article/10.3390/nano12111930/s1, Figure S1: Effect of OH-3MeOPP protonation (0.5 N HCl) on the shape of the UV-Vis spectra; Figure S2: Superposed FT-IR spectra of OH-3MeOPP and Pt-OH-3MeOPP as KBr pellets; Figure S3: Goniometric phases for OH-3MeOPP (a) and Pt-OH-3MeOPP (b); Figure S4: Linear dependence between the absorption intensity read at 457 nm for the Pt-OH-3MeOPP-AuNPs and the Pt-OH-3MeOPP; Figure S5: The emission spectra of OH-3MeOPP in acid medium (pH = 2.5) or solely in DMF, λexcitation = 418 nm, excitation slits 15 nm, emission slits 5 nm; Figure S6: Overlapped emission spectra for the detection of HQ by OH-3MeOPP in acid medium λexcitation = 418 nm, excitation slits 15 nm, emission slits 5 nm. Detailed linear dependence between the intensity of emission read at 654 nm and the HQ concentration; Figure S7: Overlapped emission spectra for the tested interfering compounds in the fluorescence detection of HQ; Table S1: The tested interfering analytes and the average percentage errors in the case of fluorimetric detection of HQ. References [103,104,105,106,107,108,109,110,111,112,113] are cited in the supplementary materials.
