# Supplementary material for: One A3B Porphyrin Structure—Three Successful Applications
Source: Nanomaterials (Basel). 2022 Jun 5;12(11):1930. doi: 10.3390/nano12111930 (PMC9182125; doi:10.3390/nano12111930)
Supplement: Supplementary file 1 [file nanomaterials-12-01930-s001.zip › nanomaterials-1742178-supplementary.pdf]

nanomaterials

Supplementary Material

One A3B Porphyrin Structure—Three Successful Applications

Ion Fratilescu [1], Anca Lascu [1], Bogdan Ovidiu Taranu [2,*], Camelia Epuran [1], Mihaela Birdeanu [2], Ana-Maria Macsim [3], Eugenia Tanasa [4], Eugeniu Vasile [4] and Eugenia Fagadar-Cosma [1,*]

[1] Institute of Chemistry "Coriolan Dragulescu", Mihai Viteazu Ave. 24, 300223 Timisoara, Romania; ionfratilescu@acad-icht.tm.edu.ro (I.F.); alascu@acad-icht.tm.edu.ro (A.L.); ecamelia@acad-icht.tm.edu.ro (C.E)
[2] National Institute for Research and Development in Electrochemistry and Condensed Matter, Plautius Andronescu Street 1, 300224 Timisoara, Romania; mihaelabirdeanu@gmail.com
[3] Institute of Macromolecular Chemistry "Petru Poni", Grigore Ghica Vodă Alley, No. 41A, 700487 Iasi, Romania; macsim.ana@icmpp.ro
[4] Faculty of Applied Chemistry and Materials Science, University Politehnica of Bucharest, Splaiul Independentei 313, Sector 6, 060042 Bucharest, Romania; eugenia.vasile27@gmail.com (E.T.); eugeniu.vasile@upb.ro (E.V.)
* Correspondence: b.taranu84@gmail.com (B.O.T.); efagadar@yahoo.com (E.F.-C.)

3.1.2. Discussion regarding UV-Vis spectra of OH-3MeOPP and Pt-OH-3MeOPP

Effect of protonation on UV-Vis spectra of OH-3MeOPP

Upon protonation with a protic acid, most of the porphyrin-bases manifest pronounced aggregation [103] and the red shift of the Soret band and changes regarding the position of Q(0,0) bands take place. Besides, in various substituted porphyrins protonation is usually leading to saddle conformation that allows aryl groups to rotate into the plane of the ring and subsequently to interact with the extended π-system [104]. It is already known that the electron densities for the e_g orbitals are distributed on the pyrrole carbon atoms, the methane bridges and the core nitrogen atoms. Instead, the a_{2u} electron densities are balanced between the methene carbons and pyrrole nitrogens [105]. The wavelengths of the Q(0,0) band of porphyrin dications are depending on the extent of π-electron donation from *meso*-substituents to the porphyrin core and in this respect, it is known that $-OCH_3$ group has a very good donor ability [106].

The changes that are displayed in the UV-Vis spectra of OH-3MeOPP in DMF-aqueous solution at different values of pH have been investigated and are presented in Figure S1.

A solution of OH-3MeOPP in DMF ($c = 7.214 \times 10^{-6}$ M) was treated with portions of 0.01 mL of HCl solution (c = 0.5 N). The UV-Vis spectra were recorded after 90 seconds stirring, and the pH of the solutions was measured.

By increasing the acidity, the protonation of the core nitrogen atoms of the OH-3MeOPP is produced. The splitting of the Soret band into two well-resolved Lorentzian bands (Figure S1) located around 417 nm and 454 nm, respectively is the proof of the existing equilibrium between the monomer and the J-type aggregate species. Reaching the pH value of 1.6, the D_{2h} symmetry of the porphyrin-base is increasing to D_{4h} corresponding to more symmetrical dication species. This increase of symmetry is associated with the decrease in the number of Q bands, to only one that is the QI band. The QI band is presenting a remarkable bathochromic shift accompanied by a significant hyperchromic effect.

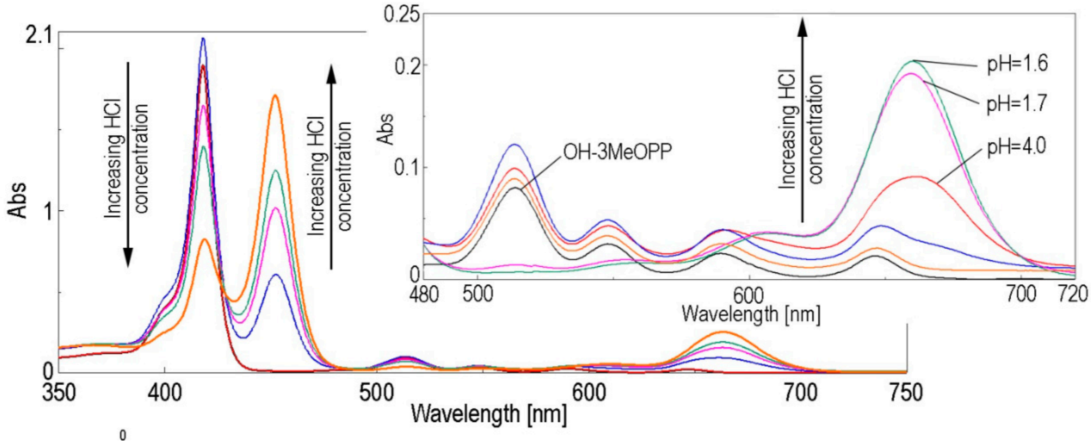

Figure S1. Effect of OH-3MeOPP protonation (0.5 N HCl) on the shape of the UV-Vis spectra.

3.1.3. Comparative aspects of OH-3MeOPP and Pt-OH-3MeOPP FT-IR spectra

The general expected features and characteristic peaks are identifiable in the FT-IR spectra of both the base- and the metalated porphyrin (Figure S2), as follows: the band located at 3734 cm^{-1} belongs to the stretching vibrations of the -OH group [107]; the symmetrical and asymmetrical stretching vibrations of the C-H bonds can be identified in the 2922 – 2856 cm^{-1} range [108]. The characteristic aromatic stretching vibration of the C=C bond can be detected at 1588 cm^{-1} [109] and the vibration of the C=N bond is noticed at 1462 cm^{-1} [110]. The medium intensity band around 1250 cm^{-1} is corresponding to aromatic C-O-C vibration. An additional feature is the band at 723 cm^{-1} representing the symmetrical methyl bending vibrations [111]. The significant difference in the FT-IR spectra, that confirms the full metalation process, is the absence of the peak located at 3314 cm^{-1} in the spectrum of Pt-OH-3MeOPP that represents the stretching vibration of the internal N-H bonds in the porphyrin-base.

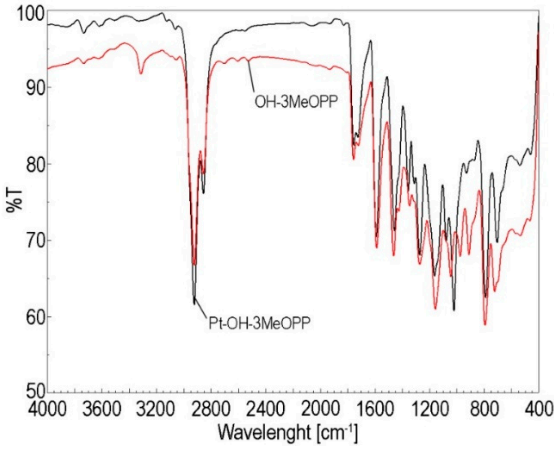

Figure S2. Superposed FT-IR spectra of OH-3MeOPP and Pt-OH-3MeOPP as KBr pellets.

3.2. Combined microscopic investigations

X-ray diffraction (XRD) spectra were registered on a Panalytical X'PERT MPD X-ray Diffractometer, in the range 2θ = 2°–80°. An X-ray beam characteristic to Cu Kα radiation was used (λ= 1.5418 Å). X-ray diffraction was employed for structural characterization of the samples involved in this study.

The X-ray diffraction patterns for OH-3MeOPP (**a**) and Pt-OH-3MeOPP are showns in Figure S3.

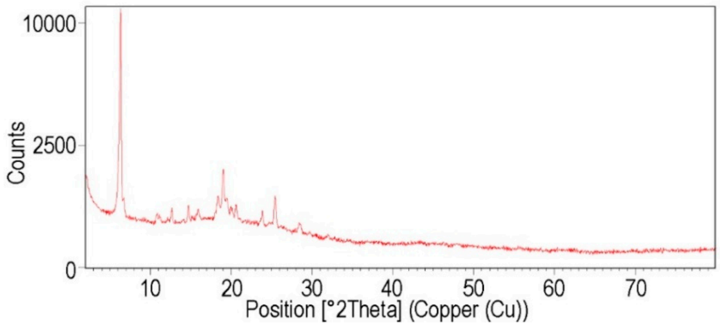

(**a**)

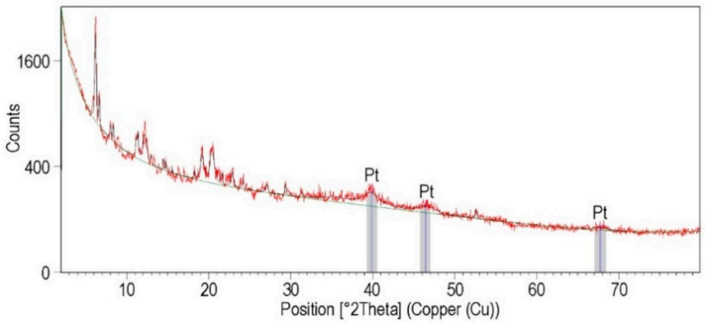

(**b**)

Figure S3. The X-ray diffraction patterns for OH-3MeOPP (**a**) and Pt-OH-3MeOPP (**b**).

The X-ray diffraction patterns shown in Figure S3a is obtained on the OH-3MeOPP sample. The diffraction pattern from Pt-OH-3MeOPP sample (Figure S3b) highlights the maxima from Figure S3a, but and the diffraction peaks with the significant 2θ degrees at 39.98°, 46.5°, and 67.87° corresponding to the Miller families of planes (111), (200), and (220) of the Pt compound, as indicated by Ref Pattern Platinum 04-001-2680.

3.3.2 Method for obtaining of the Pt-OH-3MeOPP-AuNPs complex

The dependence between the intensity of absorption read at the wavelength of the newly formed peak (457 nm) for the Pt-OH-3MeOPP-AuNPs and the Pt-OH-3MeOPP concentration (Figure S4) is linear in the concentration domain $1.98 – 4.66 \times 10^{-7}$ M.

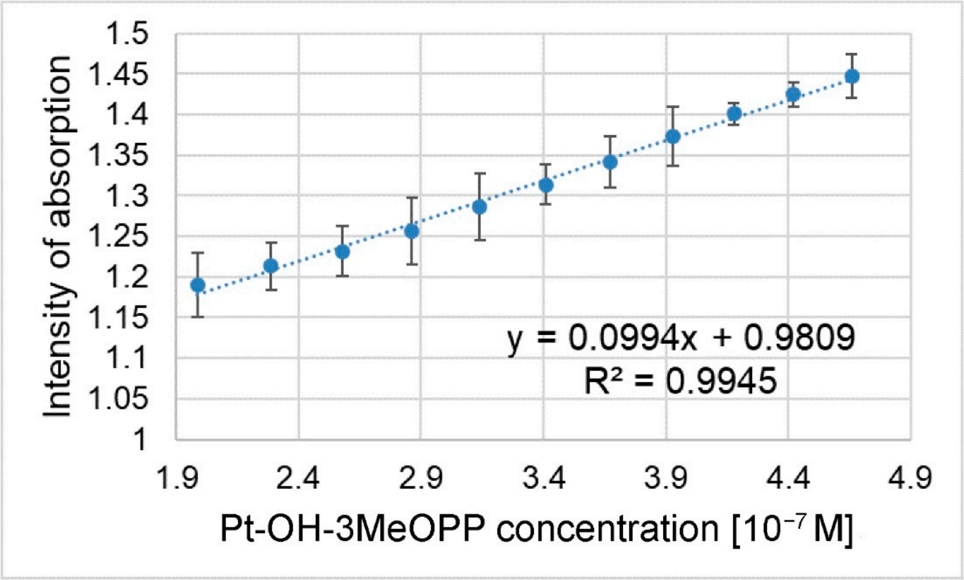

Figure S4. Linear dependence between the absorption intensity read at 457 nm for the Pt-OH-3MeOPP-AuNPs and the Pt-OH-3MeOPP.

3.4.2. The fluorescence detection of HQ by OH-3MeOPP in acid medium

Since the Pt-OH-3MeOPP-AuNPs has no fluorescence [112,113] and the attempts of fluorescent detection of HQ by OH-3MeOPP gave no straight results, we proceeded to use as sensitive material OH-3MeOPP in acidic medium.

The emission spectrum of OH-3MeOPP in DMF exhibited two maxima, a strong Qx(0,0) fluorescence band situated at 654 nm and a weaker emission band located at 730 nm, assigned to Qx(0,1) transition, having the same allure and position no matter of acidified or only organic solvent (Figure S5).

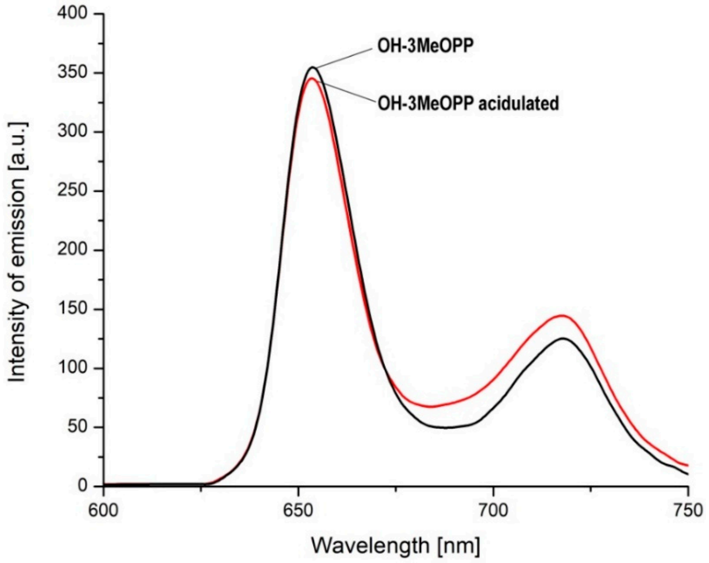

Figure S5. The emission spectra of OH-3MeOPP in acid medium (pH= 2.5) or solely in DMF, $\lambda_{excitation}$ = 418 nm, excitation slits 15 nm, emission slits 5 nm.

Method of fluorescence detection: 3 mL OH-3MeOPP solution in DMF (c = 7.214 ×10^{-6} M) were acidulated to pH-2.5 with HCl (c = 0.5 N) and portions of HQ solution in water (c = 2 × 10^{-5} M) were added. After each adding, the mixtures are stirred for 90 seconds and the emission spectra are recorded (Figure S6). The OH-3MeOPP proved to be efficient in the detection of HQ in a slightly narrower concentration domain than the UV-Vis detection, 6.578 ×10^{-8}–6.35 ×10^{-6} M, with a 99.33% confidence (Figure S6-detail). The limit of detection (LOD), calculated in the same way as for UV-Vis detection is 0.022 μM.

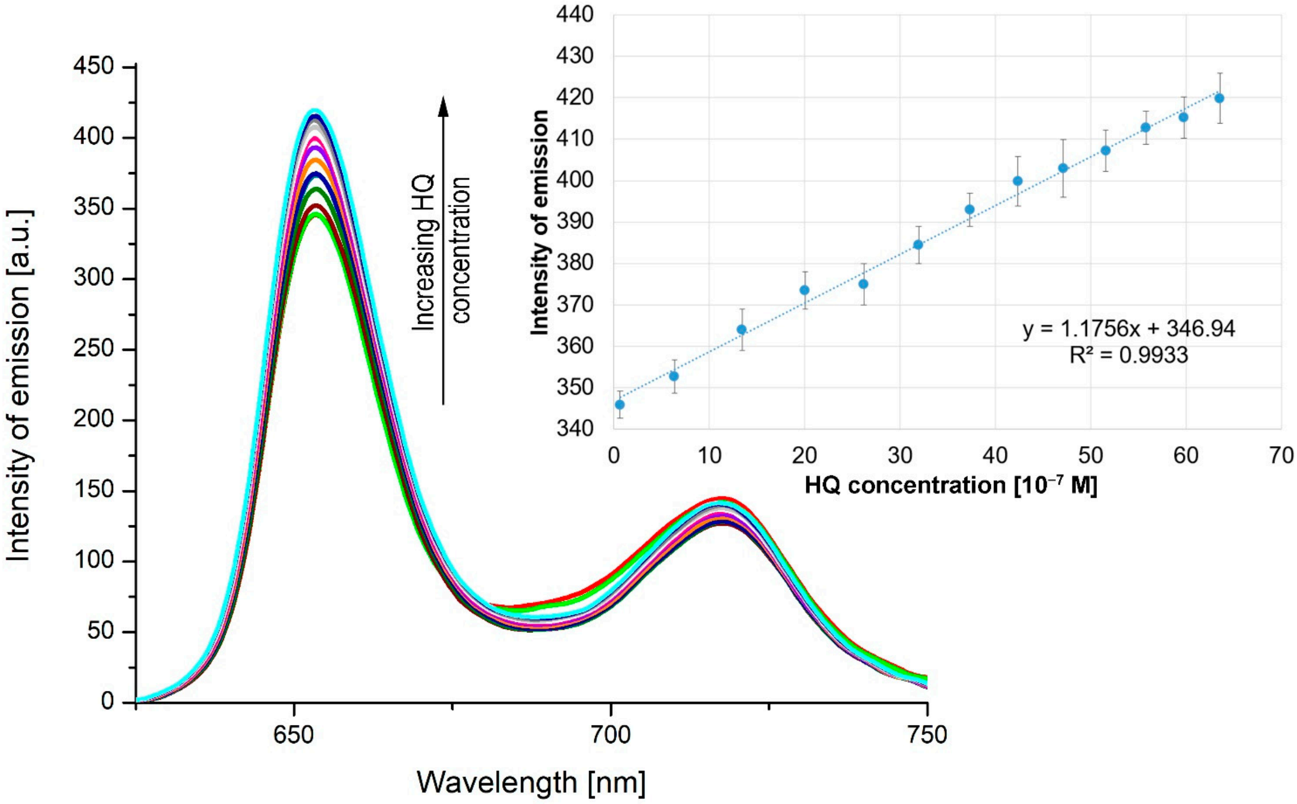

Figure S6. Overlapped emission spectra for the detection of HQ by OH-3MeOPP in acid medium $\lambda_{excitation}$ = 418 nm, excitation slits 15 nm, emission slits 5 nm. In detail linear dependence between the intensity of emission read at 654 nm and the HQ concentration.

Interference study for the fluorescent detection of HQ

A solution of OH-3MeOPP in DMF was prepared, with the concentration of 7.214×10^{-6} M and then acidulated to pH = 2.5 with HCl solution (c = 0.5 N). The reference sample consisted in 3 mL acidulated OH-3MeOPP solution in DMF, 0.06 mL HQ solution (c = 2×10^{-5} M) and 0.1 mL water. In each of the other samples, beside 0.06 mL HQ solution, a volume of 0.1 mL of each interfering analyte was added (c = 2×10^{-3} M) so that the interfering analyte concentration in the mixture should be 100-fold higher than that of HQ. The mixtures were stirred for 90 seconds and then the emission spectra were recorded and overlapped as presented in Figure S7.

The tested interfering analytes were the same as the ones used in the case of the optical detection: ascorbic acid (AA), urea, DL-menthol, calcium gluconate (CaGlu), lactic acid (LA), glucose (Glu), KCl, sodium acetate (SA), calcium lactate (CaL), $FeCl_3$, sodium salicylate (SS), NaCl, KI.

Table S1. The tested interfering analytes and the average percentage errors in the case of fluorescence detection of HQ.

Interfering analytes	Average percentage error $\|\Delta I/I\| \times 100$ [%]
Ref=HQ	0
SA	0.028433
Urea	0.056867
KCl	0.2559
$FeCl_3$	0.369633
KI	0.540233
CaL	0.739266
AA	0.9383
CaGlu	1.052033
LA	1.251066
SS	1.2795
NaCl	1.478533
DL-Menthol	2.104066
Glu	25.10662496

From table S1 it can be concluded that the only one compound that disturbs the fluorescence detection of HQ by OH-3MeOPP introducing an average error of 25%, is glucose.

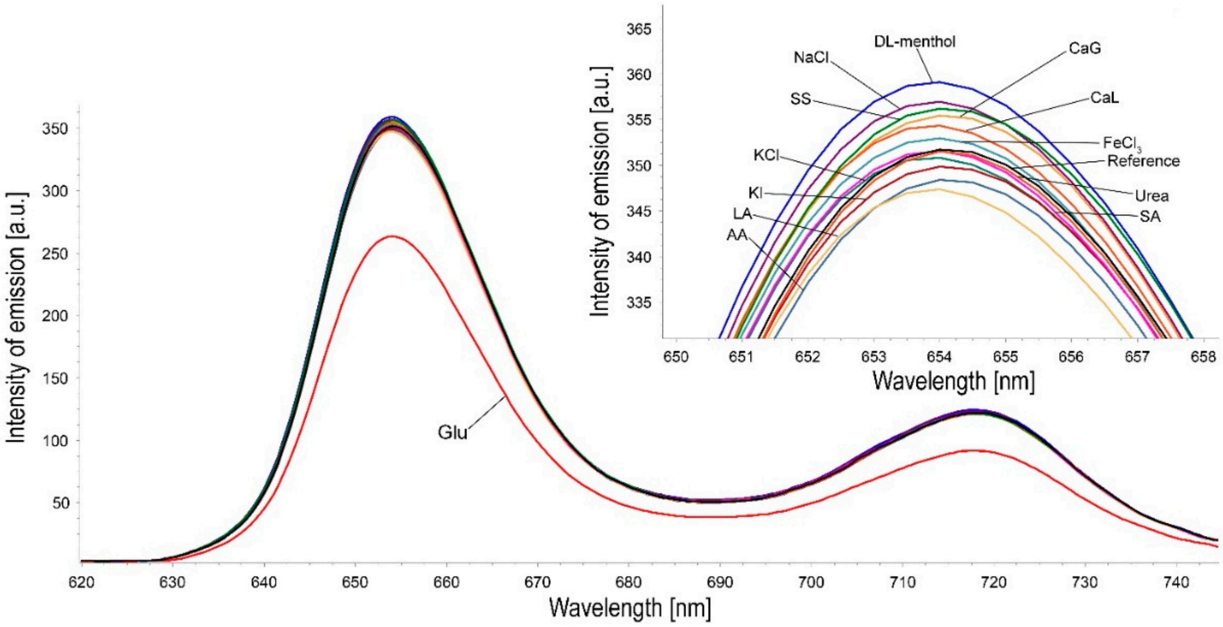

Figure S7. Overlapped emission spectra for the tested interfering compounds in the fluorescence detection of HQ.

References

103. Fagadar-Cosma, E.; Cseh, L.; Badea, V.; Fagadar-Cosma, G.; Vlascici, D. Combinatorial Synthesis and Characterization of New Asymmetric Porphyrins as Potential Photosensitizers in Photodynamic Therapy. *Comb. Chem. High T. Scr.* **2007**, *10*, 466−472. https://doi.org/10.2174/138620707781996385.
104. Senge, M.O. A conformational study of 5,10,15,20-tetraalkyl-22H+,24H+-porphyrindiium salts (dication salts). *Z. Naturforsch. B*. **2000**, *55,* 336–344. https://doi.org/10.1515/znb-2000-3-417.
105. Gouterman, M. Spectra of porphyrins. *J. Mol. Spectrosc*. **1961**, *6,* 138–163. https://doi.org/10.1016/0022-2852(61)90236-3.
106. Zakavi, S.; Omidyan, R.; Ebrahimi, L.; Heidarizadi, F. Substitution effects on the UV–vis and 1H NMR spectra of the dications of meso and/or β substituted porphyrins with trifluoroacetic acid: Electron-deficient porphyrins compared to the electron-rich ones. *Inorg. Chem. Commun*. **2011**, *14*, 1827–1832, https://doi.org/10.1016/j.inoche.2011.08.019.
107. Wang, C.; Wang, J.; Wang, J.; Wang, Z.; Chen, Z.; Li, X.; Shen, M.; Yan, W.; Kang, X. The Role of Impregnated Sodium Ions in Cu/SSZ-13 NH3-SCR Catalysts. *Catalysts* **2018**, *8*, 593. https://doi.org/10.3390/catal8120593.
108. Öztürk, N.; Çırak, Ç.; Bahçeli, S. FT-IR Spectroscopic Study of 1,5-Pentanedithiol and 1,6-Hexanedithiol Adsorbed on NaA, CaA and NaY Zeolites. *Z. Naturforsch. A*. **2014**, *60*, 633–636. https://doi.org/10.1515/zna-2005-8-913.
109. El-Tabl, A.S. Synthetic and spectroscopic investigations of some transition metal complexes of hydroxy-oxime ligand. J Chem Res. **2004**, *2004*, 19–22. https://doi.org/10.3184/030823404323000657.
110. Wang, M.Y.; Zhu, W.; Wang, Q.; Yang, Y.; Zhou, H.; Zhang, F.; Zhou, L.; Razal, J.M.; Wallace, G.G.; Chen, J. Metal porphyrin intercalated reduced graphene oxide nanocomposite utilized for electrocatalytic oxygen reduction. *Green Energy Environ*. **2017**, *2*, 285–293. https://doi.org/10.1016/j.gee.2017.06.001.
111. Zeier, J.; Schreiber, L. Fourier transform infrared-spectroscopic characterisation of isolated endodermal cell walls from plant roots: chemical nature in relation to anatomical development. *Planta* **1999**, *209*, 537–542. https://doi.org/10.1007/s004250050758.
112. Schneider, G.; Decher, G.; Nerambourg, N.; Praho, R.; Werts, M.H.V.; Blanchard-Desce, M. Distance-Dependent Fluorescence Quenching on Gold Nanoparticles Ensheathed with Layer-by-Layer Assembled Polyelectrolytes. *Nano Lett.* **2006**, *6*, 530–536. https://doi.org/10.1021/nl052441s.
113. Qin, H.; Ma, D.; Du, J. Distance dependent fluorescence quenching and enhancement of gold nanoclusters by gold nanoparticles. *Spectrochim. Acta A Mol. Biomol. Spectrosc*. **2018**, *189*, 161−166. https://doi.org/ 10.1016/j.saa.2017.08.025.
